# Joint multi-omics discriminant analysis with consistent representation learning using PANDA

**DOI:** 10.21203/rs.3.rs-4353037/v1

**Published:** 2024-05-17

**Authors:** Muhammad Aminu, Lingzhi Hong, Natalie Vokes, Stephanie T. Schmidt, Maliazurina Saad, Bo Zhu, Xiuning Le, Cascone Tina, Ajay Sheshadri, Bo Wang, David Jaffray, Andy Futreal, J. Jack Lee, Lauren A. Byers, Don Gibbons, John Heymach, Ken Chen, Chao Cheng, Jianjun Zhang, Jia Wu

**Affiliations:** 1 Department of Imaging Physics, The University of Texas MD Anderson Cancer Center, Houston, TX, USA.; 2 Department of Thoracic/Head and Neck Medical Oncology, The University of Texas MD Anderson Cancer Center, Houston, TX, USA.; 3 Department of Pulmonary Medicine, The University of Texas MD Anderson Cancer Center, Houston, TX, USA.; 4 Department of Medical Biophysics, University of Toronto, Ontario, Canada.; 5 Office of the Chief Technology and Digital Officer, The University of Texas MD Anderson Cancer Center, Houston, TX, USA.; 6 Department of Genomic Medicine, The University of Texas MD Anderson Cancer Center, Houston, TX, USA.; 7 Department of Biostatistics, The University of Texas MD Anderson Cancer Center, Houston, TX, USA.; 8 Department of Bioinformatics and Computational Biology, The University of Texas MD Anderson Cancer Center, Houston, TX, USA.; 9 Department of Medicine, Institution of Clinical and Translational Research, Baylor College of Medicine, Houston, TX, USA.

## Abstract

Integrative multi-omics analysis provides deeper insight and enables better and more realistic modeling of the underlying biology and causes of diseases than does single omics analysis. Although several integrative multi-omics analysis methods have been proposed and demonstrated promising results in integrating distinct omics datasets, inconsistent distribution of the different omics data, which is caused by technology variations, poses a challenge for paired integrative multi-omics methods. In addition, the existing discriminant analysis-based integrative methods do not effectively exploit correlation and consistent discriminant structures, necessitating a compromise between correlation and discrimination in using these methods. Herein we present PAN-omics Discriminant Analysis (PANDA), a joint discriminant analysis method that seeks omics-specific discriminant common spaces by jointly learning consistent discriminant latent representations for each omics. PANDA jointly maximizes between-class and minimizes within-class omics variations in a common space and simultaneously models the relationships among omics at the consistency representation and cross-omics correlation levels, overcoming the need for compromise between discrimination and correlation as with the existing integrative multi-omics methods. Because of the consistency representation learning incorporated into the objective function of PANDA, this method seeks a common discriminant space to minimize the differences in distributions among omics, can lead to a more robust latent representations than other methods, and is against the inconsistency of the different omics. We compared PANDA to 10 other state-of-the-art multi-omics data integration methods using both simulated and real-world multi-omics datasets and found that PANDA consistently outperformed them while providing meaningful discriminant latent representations. PANDA is implemented using both R and MATLAB, with codes available at https://github.com/WuLabMDA/PANDA.

High-throughput technologies have revolutionized the study of complex biological processes by enabling the generation of high-dimensional multi-omics datasets, with each omics technology capturing a different set of biological features that may offer orthogonal biological insights. A challenge in integrative multi-omics analysis is effectively mapping the data across omics platforms to shared entities or features. One approach to fusing the various feature sets that has proven successful is using joint feature extraction to find a common latent space that captures as much of the shared and complementary information in the original omics data as possible. Previously developed joint feature extraction approaches include unsupervised methods such as integrative nonnegative matrix factorization (intNMF)^1^, regularized generalized canonical correlation analysis (RGCCA)^2^, and probabilistic model for factor analysis (MEFISTO)^3^ and supervised approaches such as Data Integration Analysis for Biomarker discovery using Latent cOmponents (DIABLO)^4^ and Multi-Omics Graph cOnvolutional NETworks (MOGONET)^5^. These methods have all demonstrated promising results in integrating distinct omics data measured on the same samples. However, a key challenge that is not explicitly addressed by these methods is how to effectively capture biological consistency or correspondence to mine consistent structural information across omics. Since the distinct omics datasets are collected from identical samples, there is a correspondence among the various omics. For instance, in the process of protein synthesis, the genetic information encoded in DNA is transcribed into RNA. Conversely, the genetic information encoded in RNA is used to produce proteins via translation. Together, these processes represent the flow of genetic information from DNA to RNA to proteins, which is referred to as omics correspondence. The latent components identified by integrative algorithms should preserve this important property. On the other side, the current landscape of integrative multi-omics data analysis faces a crucial challenge in balancing discriminative capability and cross-omics correlation within extracted latent components. While discriminant analysis-based (supervised) methods excel in stratifying observations into distinct categories, they lack comprehensive information on cross-omics correlation. The need for integrative multi-omics methods that effectivelycapture both discriminant and correlation structures is evident, as existing approaches fall short in achieving this dual objective. Additionally, the impact of inconsistent distribution arising from technical and/or platform variations poses a significant hurdle in the performance of current integrative multi-omics methods. Addressing these limitations is imperative for the development of robust techniques that can extract biologically and clinically relevant biomarkers, striking a balance between discrimination and correlation in the context of integrative multi-omics analysis.

Herein we present a novel multi-omics discriminant analysis method, PAN-omics Discriminant Analysis (PANDA), that jointly integrates multi-omics data and addresses the above-mentioned challenges. PANDA learns joint discriminant representations of paired omics data with representation consistency, which helps overcome technical variations that affect the existing integrative methods. Through optimizing a novel objective function that captures omics consistency while simultaneously maximizing both the cross-omics correlation and discrimination between omics, PANDA latent representations can capture maximum cross-omics correlations and discriminant information, overcoming the need to compromise between discrimination and correlation, and does not rely on prior knowledge of the relationships between the omics data to perform integration. In addition, PANDA obtains omics-specific latent components, which is highly desirable in integrative multi-omics analysis. It has been previously reported that methods that determine omics-specific components often had performance superior to that of methods designed for finding shared or mixed components^6^.

In summary, PANDA meets the challenges of paired multi-omics data integration through a novel objective function that 1) explicitly incorporates consistency representation learning to minimize the differences among omics, resulting in effective and efficient handling of technical variations; 2) fuses multi-omics consistency and cross-omics correlation to model the common properties among omics, which can lead to more robust latent representations; 3) determines joint discriminant and consistent latent representations that maximally capture both the correlation and discriminant structure between omics; and 4) constrains the omics-specific latent representations to be uncorrelated with minimum redundancy, which is highly desirable in multi-omics integration.

PANDA can be used for different kinds of analysis, including joint discriminant analysis (joint dimensionality reduction), complex multi-omics data integration, differential marker gene identification, multi-omics data denoising, and estimating correlations among cross-omics features (e.g., correlations between genes and proteins). We demonstrated the advantages of PANDA with both simulated and real-world multi-omics datasets. Regarding simulated data, we show that PANDA effectively finds a discriminant common latent space for the different omics data in which class patterns are maximally separated while within-class variations are minimized. In addition, the PANDA latent representations are robust against increased noise and sparsity levels while preserving structural correspondence between the different omics. Regarding real-world multi-omics datasets, the PANDA latent components capture both joint discriminant structures and cross-omics relationships, which contrasts with methods that extract features based solely on sample covariance or heterogeneity. Furthermore, we demonstrate that PANDA latent components are not redundant and enable clinical outcome prediction. Finally, using breast cancer and single-cell multi-omics peripheral blood mononuclear cell (PBMC) datasets, we show that the PANDA latent components recover important genes that enable robust biomarker identification, and that PANDA outperforms several state-of-the-art (SOTA) multi-omics data integration methods.

## Results

### PANDA algorithm

PANDA is a joint discriminant analysis method aimed at fusing multi-omics datasets through finding a discriminant common latent space. PANDA captures cross-omics interaction and consistency and uses an uncorrelated constraint to ensure that the extracted latent components for each omics (omics-specific components) are not highly correlated (i.e., do not capture redundant information). Because the components extracted using PANDA contain valuable discriminant information, we refer to them as discriminant components. These components can be used as inputs to several multi-omics analysis tools to enable efficient, improved downstream analysis. We demonstrated the advantages of PANDA over other integrative multi-omics methods through four distinct downstream analyses: single-cell multi-omics data visualization, patient (or tumor) classification, biomarker identification, and clinical outcome prediction. We focused on the application of PANDA to single-cell and bulk sequencing data; however, PANDA is platform-agnostic and readily applicable to other multi-omics datasets. PANDA is implementable as an R package and is freely available at https://github.com/WuLabMDA/PANDA.

### Systematic benchmarking with simulated single-cell multi-omics data demonstrates superior performance of PANDA over SOTA approaches

Initially, we benchmarked PANDA against several other SOTA multi-omics integration approaches using simulated single-cell multi-omics data. The datasets (paired single-cell RNA sequencing [scRNA-seq] and single-cell sequencing assay for transposase-accessible chromatin [scATAC-seq]) were simulated using the MOSim R package^7^. MOSim mimic the properties of single-cell multi-omics data, such as noise and high sparsity. We assessed PANDA in terms of discriminant latent component recovery/estimation, in other words, how well it found a common latent space that best captures the discriminant structure in the data, representation consistency, and whether high noise and high sparsity levels affect PANDA performance. For comparison, we considered nine other SOTA multi-omics integration approaches listed in Table 2: MEFISTO^3^, multi-omics factor analysis (MOFA)^8,9^, DIABLO^4^, RGCCA^2^, intNMF^1^, multiple co-inertia analysis (MCIA)^10–12^, joint and independent variation explained (JIVE)^13^, tensorial independent component analysis (tICA)^14^, and iCluster^15^. We compared the performance of the different approaches based on how well similar samples were grouped and dissimilar samples were separated in the subspaces determined using the different benchmarked approaches. Specifically, we applied a consensus clustering algorithm to the 2D latent spaces determined using these approaches and measured the agreement among the obtained cluster labels and ground-truth labels using the adjusted Rand index (ARI) and normalized mutual information (NMI) metrics.

**Extended Data Fig. 1 |:**
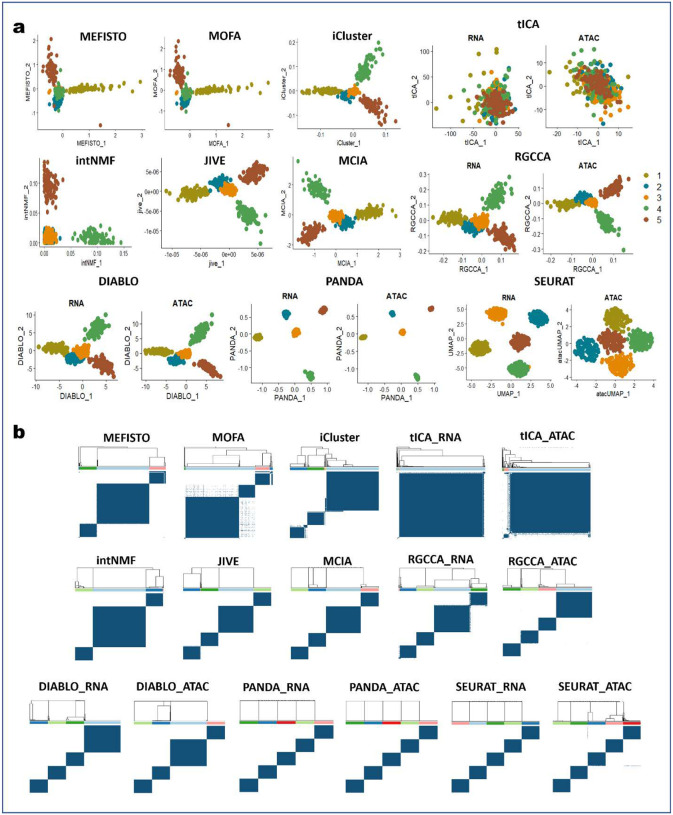
Benchmarking PANDA with SOTA multi-omics integration methods using a simulated single cell dataset. **a,** 2D projections of the different multi-omics integration approaches. **b,** Consensus clustering heat maps for the different approaches. The results for all compared multi-omics methods in our analysis are shown.

**Fig. 1 |:**
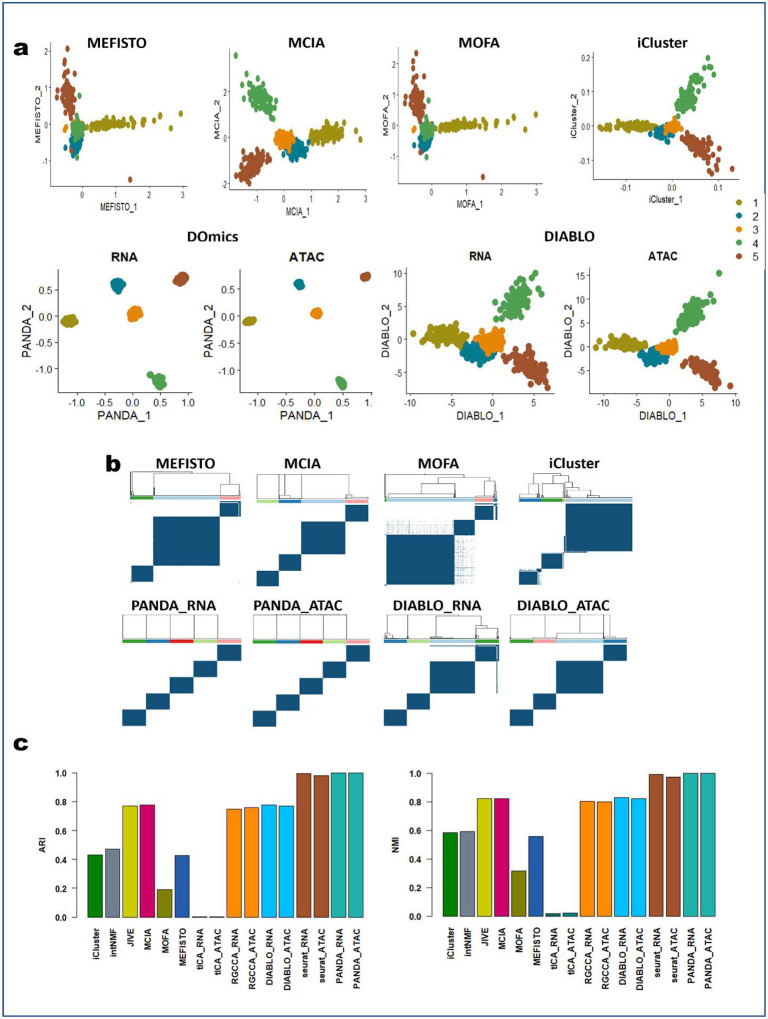
Benchmarking PANDA with SOTA multi-omics data integration methods using simulated multi-omics dataset. **a**, 2D projections of the different integrative multi-omics approaches. **b**, Consensus clustering heat maps for the 2D projections of the different compared approaches. **c**, Bar plots of the clustering performance (ARI and NMI values) obtained when applying hierarchical clustering to the 2D projections of the different approaches.

First, we compared 2D visualizations of cells obtained using the different approaches (Fig. 1a and Extended Data Fig. 1a). Worth noting is that some of the approaches (MEFISTO, MOFA, iCluster, intNMF, JIVE, and MCIA) are designed to determine single latent spaces for the different omics data (scRNA-seq and scATAC-seq), whereas tICA, RGCCA, DIABLO, and PANDA are designed to determine multiple (omics-specific) latent spaces common to all omics data. Thus, we showed one 2D latent space each for MEFISTO, MOFA, iCluster, intNMF, JIVE, and MCIA, and two 2D latent spaces (for the scRNA-seq and scATAC-seq, respectively) each for tICA, RGCCA, DIABLO, and PANDA. An effective integration algorithm should determine the cell embeddings where biological variation is faithfully conserved, and dissimilar cell types are well separated. PANDA yielded better and more accurate 2D representations of the data than did the other approaches. We found that PANDA obtained common latent spaces where cells of the same type were projected close to each other whereas dissimilar cell types were maximally separated. PANDA also demonstrated clearer patterns in the common latent spaces, with cells being aggregated according to their types. This is further validated by the corresponding consensus matrices for the PANDA algorithm (Fig. 1b and Extended Data Fig. 1b), where very clean cluster partitions (five clusters) are present, with cells having very high consensus (with the dark blue color indicating cells always clustered together for 100 subsampling attempts). Moreover, we observed that PANDA was robust to high levels of noise and sparsity in the datasets, as it performed well in determining faithful latent representations of the datasets by separating dissimilar cells in both the scRNA-seq and scATAC-seq latent spaces.

Owing to the consistency representation term in PANDA, the scRNA-seq and scATAC-seq common latent spaces determined using PANDA have similar data patterns, an indication of the extent to which biological variation (and/or patterns) are shared across the two omics data. In contrast, iCluster, JIVE, MCIA, RGCCA, and DIABLO failed to separate clusters 2 and 3 (Fig. 1a) in 2D representations. This inferior performance can be seen in the corresponding consensus matrices (Fig. 1b and Extended Data Fig. 1b), which have only four clean partitions of the cells. More components are needed with these approaches to provide a clean partition of the datasets into five separate clusters. We observed a similar problem with the latent representations provided by MEFISTO, MOFA, and intNMF, with clusters 2,3, and 4 tending to be mixed up in the latent spaces obtained using MEFISTO and MOFA and clusters 1, 2, and 3 being mixed up in the latent space obtained using intNMF. We found that tICA performed the worst, failing to separate any of the five clusters in the datasets. The poor performance of tICA may be a result of information loss while transforming the omics datasets into correlation-of-correlation matrices to construct the tensors needed by tICA.

In terms of capturing representation consistency across the scRNA-seq and scATAC-seq data, we found DIABLO to be the only approach besides PANDA that performed well in obtaining latent spaces with similar data patterns. The latent spaces obtained using DIABLO had similar data patterns (Fig. 1a and Extended Data Fig. 1a), indicating that DIABLO can allow to some extent for sharing of evidence across omics in this benchmarking analysis. Additionally, we found that the high levels of noise and sparsity had little effect on DIABLO, RGCCA, MCIA, and JIVE, as they captured most of the discriminant structures in the original data. Meanwhile, MEFISTO, MOFA, intNMF, iCluster, and tICA were affected by the high noise and sparsity levels, with tICA affected the most.

Additionally, we used the ARI and NMI metrics to quantitively evaluate the performance of the different benchmarked integrative multi-omics approaches in determining discriminant latent spaces where the different cell types are well separated. Specifically, we compared the agreement between the ground-truth labels and obtained clustering labels by applying a consensus clustering algorithm to the latent spaces obtained using the different approaches. Fig. 1c shows the quantitative assessment of the different benchmarked methods in terms of ARI and NMI metrics. PANDA had the best clustering performance for both the scRNA-seq and scATAC-seq common latent spaces, with both an ARI and NMI value of 1.00, indicating optimal (100%) agreement between the ground-truth labels and obtained clustering labels. JIVE, MCIA, RGCCA, and DIABLO performed reasonably well, with ARI and NMI values of almost 0.8. In particular, RGCCA and DIABLO had similar performance regarding both the scRNA-seq and scATAC-seq common latent spaces, perhaps owing to a close connection between these two methods, as DIABLO is derived from the RGCCA method via incorporation of class label information. iCluster and intNMF, which were originally designed for clustering, were previously reported^6^ to perform exceptionally well, with ARI values of about 1.00 for dense, simulated multi-omics datasets. The inconsistent performance of these methods in our benchmarking study indicates their poor ability to handle single-cell multi-omics datasets with high sparsity and noise levels.

Taken together, the simulation results described above demonstrated that PANDA can be applied to noisy, complex multi-omics datasets to extract latent components that best capture the discriminant structures of the datasets. In addition, the unique capability of PANDA regarding joint discriminant analysis and consistency representation of multi-omics data further enhances latent representations of the datasets by finding multiple common latent spaces that can have similar discriminant structures, which helps handle technical variations across different modalities of the data.

### PANDA exhibits accurate, robust performance with real-world single-cell multi-omics data

Next, we demonstrated the performance of PANDA with real-world single-cell multi-omics datasets. Specifically, we used PANDA to integrate two real-world single-cell multi-omics datasets: 1) data on three cancer cell lines (HTC, HeLa, and K562)^16^ containing paired measurements of gene expression (scRNA-seq) and chromatin accessibility (scATAC-seq) for a total of 206 cells and 2) PBMC multiome data from 10x Genomics containing paired measurements of gene expression and chromatin accessibility for 11,909 cells generated from isolated nuclei of granulocytes removed via cell sorting. We presented in this section, the analysis of the cancer cell line datasets, and the analysis of the PBMC multiome dataset is presented in the next section.

**Extended Data Fig. 2 |:**
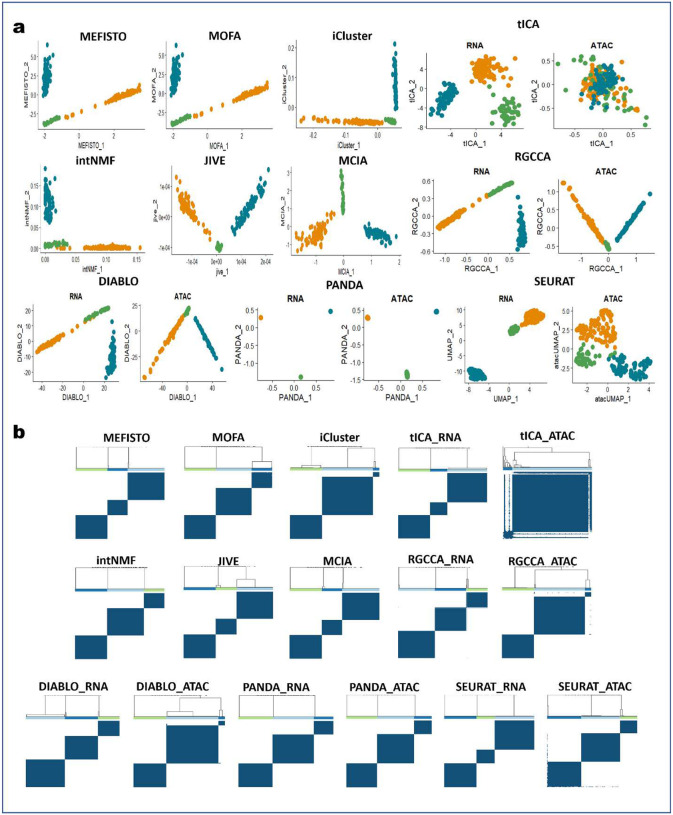
Benchmarking PANDA with SOTA multi-omics integration methods using a cancer cell line dataset. **a,** 2D projections of the different multi-omics approaches. **b,** Consensus clustering heat maps of the different approaches. The results for all compared multi-omics methods in our analysis are shown.

**Extended Data Fig. 3 |:**
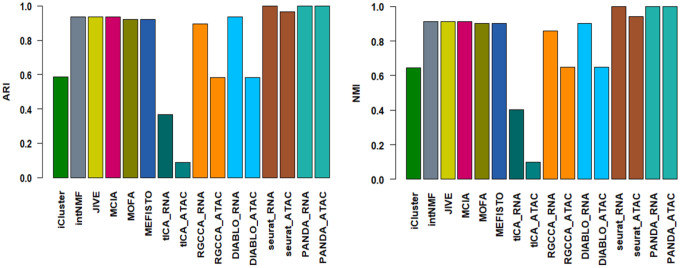
Bar plots of the performance in terms of ARI and NMI obtained by comparing the ground-truth labels for the cancer cell line data and predicted labels obtained by applying hierarchical clustering to 2D projections of the compared multi-omics integration methods.

We extracted discriminant latent components using PANDA and performed consensus clustering on the first two latent components to examine how well these components captured the discriminant structures in the datasets. We used the ARI and NMI metrics to quantitively evaluate the performance of the first two extracted latent components in distinguishing the three cancer cell lines. We further compared the performance of PANDA using these datasets with the benchmarked algorithms used in the simulation benchmarking study described above together with the well-known Seurat approach. Of note, Seurat does not provide latent components as outputs as do the other integrative multi-omics methods. Thus, we only employed the standard Seurat preprocessing approach of normalization, dimensionality reduction (using principal component analysis [PCA]), and feature embedding (using uniform manifold approximation and projection [UMAP]) with both the scRNA-seq and scATAC-seq modalities followed by consensus clustering on the UMAP embedding subspaces. Inspection of the 2D representations of the different methods (Extended Data Fig. 2a) revealed that PANDA effectively provided better grouping of the cells according to cell line of origin than did the other methods. This observation was further confirmed by consensus clustering matrices (Extended Data Fig. 2b), with PANDA exhibiting very clean grouping of the cells into three groups. In addition, PANDA obtained common latent spaces (for both scRNA-seq and scATAC-seq modalities) that have similar data structures, indicating its ability to model the common properties of the scRNA-seq and scATAC-seq modalities. The latent components from PANDA yielded meaningful correspondence of the characteristics of the scRNA-seq and scATAC-seq modalities, enabling removal of technological variations in the modalities as well as enhancing discriminant structures. Furthermore, we quantitatively compared PANDA with the other methods in terms of ARI and NMI scores and found PANDA to be the best performing method, with ARI and NMI values of exactly 1.00 on all latent spaces (Extended Data Fig. 3). Notably, tICA, RGCCA, and DIABLO seemed to be affected by the high sparsity level of the scATAC-seq modality. These methods failed to effectively group the cells according to cell line of origin in the scATAC-seq latent spaces (Extended Data Fig. 2a and 2b), resulting in ARI and NMI values lower than 0.6 for RGCCA and DIABLO and values close to zero for IICA (Extended Data Fig. 3).

**Fig. 2 |:**
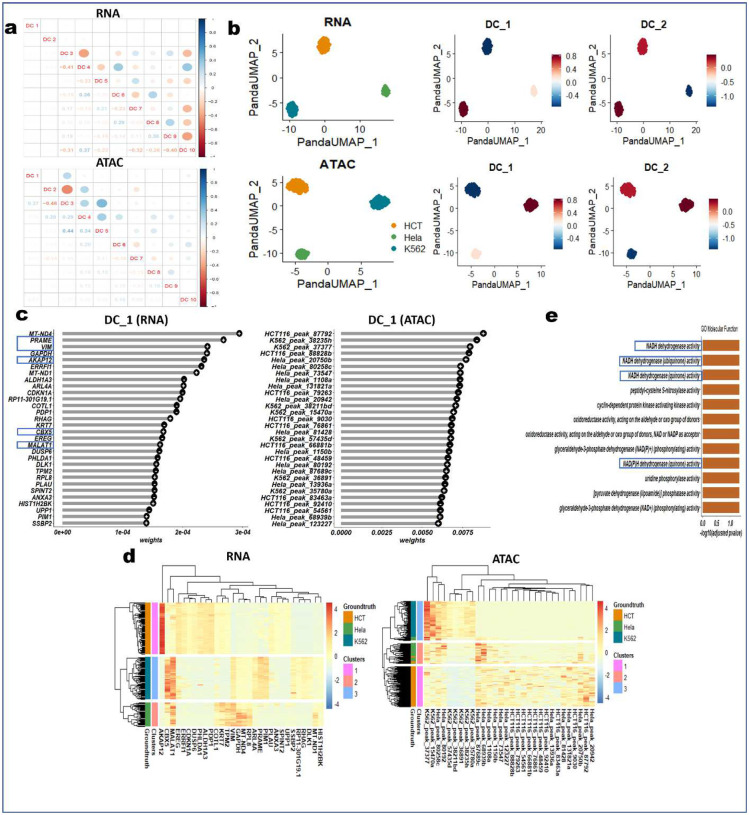
PANDA performs well in discriminating cancer cell lines of origin using single-cell multi-omics data. **a,** Correlation structures within omics for the top 10 components. **b**, 2D UMAP embedding of PANDA-extracted components (left column) and the components’ expression in the lower dimensional subspace (middle and right columns). The colors indicate the inferred components’ values. **c**, The genes and ATAC peaks with the largest absolute weights (x-axis) on PANDA latent component 1. The symbols in the black dots on the right indicate whether the weights were positive/negative. **d**, Hierarchical clustering heat maps based on the top 30 genes and peaks (z-scores) in **c**. The rows represent the three cancer cell lines (HCT, HeLa, and K562), and the columns represent the 30 genes and peaks with the largest weights on PANDA latent component 1. **e**, GO terms identified using the 30 genes in **c**.

Examining the extracted PANDA latent components further, we found that these omics-specific components were not heavily correlated, indicating they captured independent information (Fig. 2a). The uncorrelated constraint incorporated into the objective function of PANDA helps ensure that the extracted latent components are not correlated, thereby minimizing redundancy among the extracted latent components of each modality. PANDA-extracted latent components can also be used as inputs to nonlinear manifold learning approaches such as t-distributed stochastic neighbor embedding^17^ and UMAP^18^ to inspect variations captured by each component as well as for other downstream analyses, such as cell lineage/trajectory inference. We embedded cells (defined based on the 10 PANDA latent components) into lower dimensional UMAP subspaces and examined the component variabilities by visualizing the contribution of each component in the HeLa, K562, and HCT cancer cell lines. Both components 1 and 2 explained variation in three cell lines (Fig. 2b), capturing differences in these groups. Of note, component 1 (for both scRNA-seq and scATAC-seq modalities) assigned negative values (about −1), positive values (about 1), and values close to zero to the HCT, K562, and HeLa cells, respectively. In comparison, component 2 assigned negative values (about −1.5), positive values (about 0.5), and values close to zero to the HeLa, K562, and HCT cells, respectively. Overall, these two latent components captured variabilities in all three cancer cell lines.

To further characterize the molecular process, we identified genes and peaks with the greatest enrichment (absolute weight) in individual latent components by examining the PANDA projection matrix. The 30 genes and peaks with the largest absolute weights on component 1 (Fig. 2c) captured differences in expression signatures that distinguished the three cancer cell lines. PANDA identified and assigned larger weights (greater importance) to the most discriminating genes and peaks in this dataset. Heat map of the clustering analysis using these 30 genes and peaks on the first PANDA latent components optimally highlighted clusters corresponding to the three cancer cell lines while depicting the sparsity commonly seen in scRNA-seq and scATAC-seq datasets (Fig. 2d). These 30 genes collectively yielded ARI and NMI values of 1.00, and these peaks collectively yielded ARI and NMI values of 0.84 and 0.80, respectively. Furthermore, the heat maps show the expression levels for the top genes and peaks (selected on the first PANDA latent components) and in which cell lines they were highly expressed. For example, the HCT cell line was the only one exhibiting high expression of the AKAP12 gene, which was previously reported to be highly expressed in HCT-116 cells^19,20^. Likewise, hypermethylation of the AKAP12 gene was found to be high in colorectal cancer patients^21^, indicating its potential for use as a marker for monitoring the progression or carcinogenesis of colorectal cancer. Two other genes with high expressions levels in the K562 and HeLa cell lines were CBX5 and MALAT1. CBX5 was reported to had higher expression in high than in low c-Kit leukemia stem cell populations, and elevated CBX5 expression was associated with increased expression of RBMX and RBMXL1, which maintain the chromatin state essential for the survival of acute myeloid leukemia cells^22^. Similarly, it was reported that MALAT1 knockdown in HeLa cells led to alteration of the alternative splicing of pre-mRNAs and cell death^23^. In addition, PANDA latent component 1 had high positive weights for PRAME and VIM, which are associated with progression of chronic myeloid leukemia (CML)^24–26^ and acute myeloid leukemia^27,28^, respectively. When associated with BCR-ABL, PRAME expression is a marker for acute leukemia in CML patients, for whom therapeutic outcomes are generally poor^24,26^. Knockdown of PRAME in K562 cells treated with imatinib was markedly associated with an increase in apoptosis and consequent decrease in cell viability^24^. A similar study^26^ demonstrated a significant association between PRAME expression and nilotinib failure in chronic-phase CML patients. These findings identified PRAME as an attractive target for CML therapy.

Fisnally, we used the 30 genes with the largest weights for PANDA latent component 1 to identify biological processes (Fig. 2e) using g:Profiler^29^. These genes indicated enrichment of several Gene Ontology (GO) molecular functions, including NADH dehydrogenase activity (GO:0003954; P=2.99e-3), NADH dehydrogenase (ubiquinone) activity (GO:0008137; P=2.99e-3), NADH dehydrogenase (quinone) activity (GO:0050136; P=2.99e-3), and NAD(P)H dehydrogenase (quinone) activity (GO:0003955; P=2.99e-3), which were mostly associated with CML. For example, Ren et al.^30^ reported that a decrease in mitochondrial NADH and blockade of the malate-aspartate shuttle in malic enzyme 2-depleted K562 cells may lead to enhanced cell death. In addition, decreased availability of NADH may hinder the ability to maintain a sufficiently high NAD(P)H ratio, thus affecting processes such as nucleic acid synthesis and reductive biosynthesis of fatty acids and cholesterol needed to promote cell proliferation.

### PANDA identifies important biomarkers for disease-relevant monocytes

PANDA can leverage its joint discriminant analysis and correspondence structure modeling to detect and assign larger importance (weights) to differentially expressed features between two or more groups of cells while adjusting for noise and other technical issues, such as technological variations. To evaluate PANDA as a framework for identifying differentially expressed and important features in the common scenario of multi-omics and multiple cell types, we applied PANDA to the PBMC multiome (scRNA-seq and scATAC-seq) data containing 19 different cell types to identify important Differentially expressed genes associated with disease-relevant monocytes.

**Extended Data Fig. 4 |:**
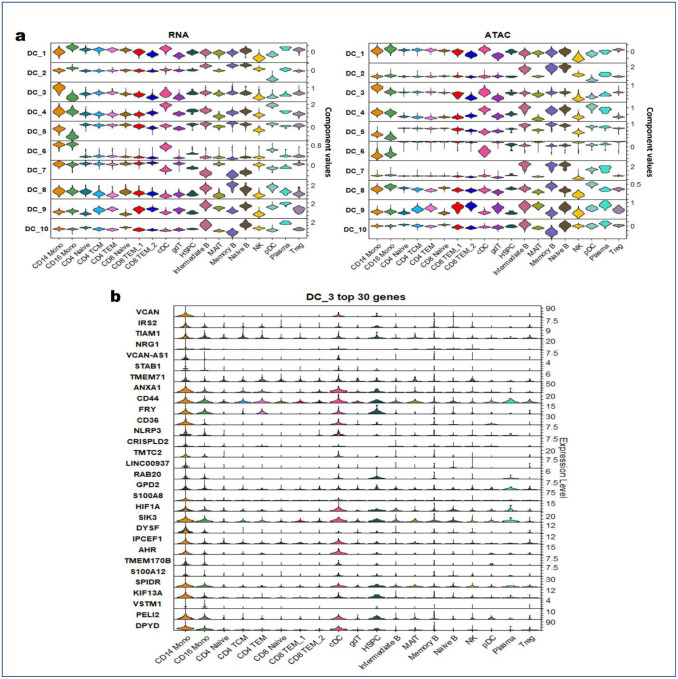
Application of PANDA to single-cell PBMC multi-omics data **a,** Violin plots of the variation in the first 10 PANDA latent components across the different cell types for the RNA (left) and ATAC (right) modalities. **b,** Violin plot of the variation in the 30 genes with the largest weights on PANDA latent component 3.

**Fig. 3 |:**
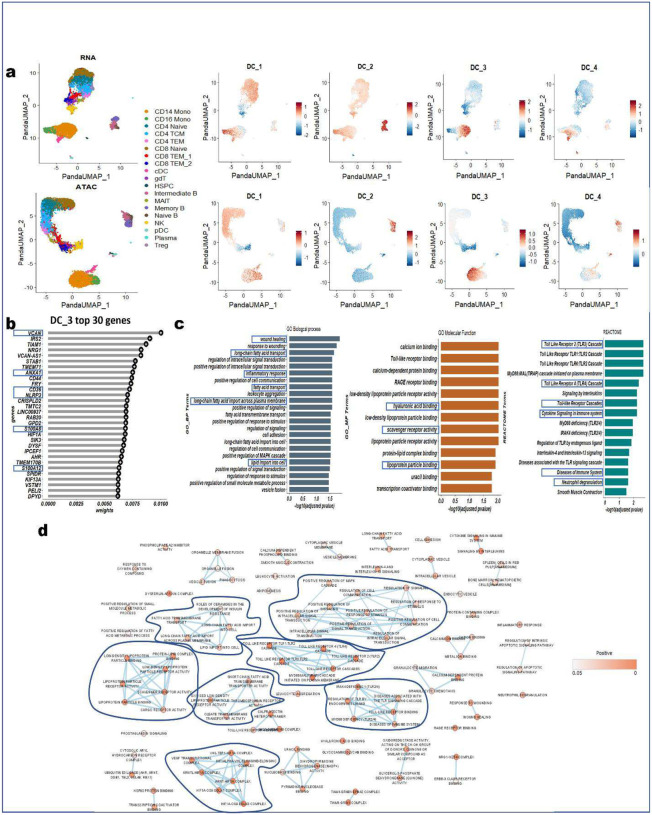
Application of PANDA to single-cell PBMC multi-omics data. **a,** 2D UMAP embedding of PANDA-extracted components (column 1) and the components’ expression in the lower dimensional subspace (columns 2–5). The colors indicate the inferred components’ values. **b**, The genes with largest absolute weights on PANDA latent component 3 (x-axis). The symbols inside the black dots on the right indicate the sign of the weights. **c,** GO terms (biological process and molecular function) and REACTOME pathways identified using the top 30 genes in **b. d**, Enrichment maps of the GO processes and REACTOME pathways in **c.** Similar pathways are connected using lines, and the line thickness indicates the extent of gene overlap between pathways.

We first extracted latent representations of the cells using PANDA and used these representations as inputs to the UMAP algorithm to determine low-dimensional embeddings depicting contributions of the PANDA latent components to the different cell types. PANDA provides a descriptive representation of these data, as inspection of the latent spaces (Fig. 3a) highlights the association of PANDA latent components in the latent spaces with cell types. The first four latent components (Fig. 3a and Extended Data Fig. 4a) had diverse contributions across the different cell types, indicating that they are robust in capturing independent information. For example, PANDA latent component 1 has a relatively large contribution (component values) on all the different cell types (on both the RNA and ATAC latent spaces) except on natural killer and CD8 TEM_2 cells, for which it has a relatively low contribution (Fig. 3a). Similarly, PANDA latent component 2 had a larger contribution on all cell types except plasmacytoid dendritic cells (in the RNA latent space) but a smaller contribution on all cell types except the B cell groups including Intermediate B, memory B, and naïve B cells (Fig. 3a and Extended Data Fig. 4a). Regarding PANDA latent component 3, it captured variations (on both the RNA and ATAC modalities) that differentiate monocytes from the rest of the cell types. To further characterize the molecular process, we investigated the genes with large absolute weights on this latent component (Fig. 3b). PANDA latent component 3 captured gene expression signatures significantly linked with inflammation, proliferation, and regulation, including pathways related to diseases of the immune system (Fig. 3c). At the same time, the top 30 genes with the largest weights on latent component 3 had positive weights, indicating upregulation of these genes along the latent component and consequently indicating higher expression of these genes in monocytes than in the rest of the cell types (Extended Data Fig. 4b). In addition, we observed that the CD14 monocytes, CD16 monocytes, and conventional dendritic cells were co-located in the latent spaces after integration as evidenced by the shared expression of key marker genes, such as CD44, SIK3, and DPYD (Extended Data Fig. 4b).

VCAN is one of the genes with the largest weights on PANDA latent component 3 and was very highly expressed in CD14 monocytes. The VCAN gene is a member of the aggrecan/versican proteoglycan family. The protein encoded is a large chondroitin sulfate proteoglycan and is a major component of the extracellular matrix. This protein is involved in cell adhesion, proliferation, migration, and angiogenesis and plays a central role in tissue morphogenesis and maintenance. Accumulation of versican facilitates wound healing (GO:0042060) by enhancing fibroblast proliferation and myofibroblast differentiation and transforming growth factor-β-mediated signaling through extracellular matrix molecule and hyaluronic acid binding (GO:0005540)^31^. CD14 monocytes (proinflammatory M1 macrophages) and CD16 monocytes (anti-inflammatory M2 macrophages) play a key role in wound healing and tissue regeneration across four-stage progression consisting of hemostasis, inflammation, proliferation, and remodeling^32^. ANXA1 (annexin A1) is another gene that is highly expressed in both CD14 monocytes and conventional dendritic cells. Annexin A1 is known to be an anti-inflammatory factor that plays a role in glucocorticoid-mediated downregulation of the early phase of the inflammatory response (GO:0050727)^33^. It also promotes chemotaxis of granulocytes and monocytes via activation of the formyl peptide receptors, enhances resolution of inflammation and wound healing, and magnifies the release of CXCL2^34–36^. For the CD36 gene identified according to mRNA expression features, several GO terms related to the lipid metabolism process were significantly enriched, including long chain fatty acid transport (GO:0015909), fatty acid transport (GO:0015908), long-chain fatty acid import across the plasma membrane (GO:0015911), lipid import into cells (GO:0140354), scavenger receptor activity (GO:0005044), and lipoprotein particle binding (GO:0071813). Monocyte adhesion, differentiation into macrophages, and polarization into proinflammatory and anti-inflammatory subsets are involved in normal and abnormal pathophysiology, including the development of atherosclerosis^37^. Monocytes contribute to the pathogenesis of atherosclerosis by pre-accumulating lipids and transporting them into atherosclerotic lesions and expressing scavenger receptors, which are exposed to hypercholesterolemic conditions in the disease state^38^.

Several of the genes with large weights on PANDA latent component 3 were previously reported to be associated with several diseases (relating to the Diseases of Immune System and Toll Like Receptor Cascade pathways in the REACTOME database) (Fig. 3c). For example, excessive NLRP3 activation in monocytes was reported to cause neuronal hypoactivity in Alzheimer disease and perioperative neurocognitive disorder^39^. Similarly, hyperactivation of monocytes in mild cognitive impairment patients contributed to the progression of Alzheimer disease^40^. In mild cognitive impairment and very early-stage Alzheimer disease patients, monocytes have been suggested to be the sources of increased oxidation products, exhibiting significantly increased chemotaxis and cytokine production in response to Toll-like receptor 2 and 4 stimulation. This increase appears to be mediated through activation of the p-p42/p44 signaling molecule mitogen-associated protein kinase (MAPK) as well as the transcription factor nuclear factor-κB^40,41^.

Moreover, the monocyte marker genes S100A8 and S100A12 were enriched with larger weights on PANDA latent component 3 and were reported to exert proinflammatory functions in Kawasaki disease (KD) patients. In particular, the S100A12 gene was reported to be highly expressed in acute KD patients^42,43^, and the plasma levels of S100A12 were reported to be elevated in patients before being given intravenous immunoglobulin (IVIG) and to decrease quickly after this therapy^44,45^. Thus, downregulation of the S100A12 gene after IVIG therapy can benefit patients by minimizing the aggregation of monocytes at inflammatory sites and preventing coronary aneurysms^44^. On the other hand, persistent elevation of the plasma levels of S100A8 after IVIG therapy was reported to be associated with increased risk of coronary aneurysms in KD patients^44^. This indicates that persistently high expression of S100A8 may be caused by prolonged survival of activated monocytes and that failure of IVIG therapy to suppress S100A8 expression in monocytes may result in continued aggregation and stimulation of monocytes at inflammatory sites^44^. Overall, IVIG therapy suppresses activated peripheral blood monocytes in KD patients by downregulating several functional genes, including S100A8^44^. Several studies have established some common pathways involved in KD that were also significantly enriched in our analyses, including RAGE receptor binding (related gene: S100A8 [GO:0050786]), disease of immune system (related genes: CD36 and S100A8), and neutrophil degranulation (related genes: CD44, CD36, CRISPLD2, and S100A8)^46,47^. More recently, Ren et al.^48^ reported an association of high expression of the S100A8 gene in monocytes with COVID-19. In particular, they reported that systemic upregulation of S100A8 in peripheral blood monocytes may contribute to cytokine storms often seen in severe COVID-19 patients. Similarly, Silvin et al.^49^ reported immense release of S100A8 calprotectin followed by changes in monocyte and neutrophil subsets in severe COVID-19 patients.

We further examined an enrichment map of the biological processes significantly enriched along the top 30 marker genes on PANDA latent component 3. The map identified seven major clusters (Fig. 3d), with the major cluster consisting of biological processes involved in the regulation of components such as positive regulation of signaling (GO:0023056; P=1.85e-2), positive regulation of the MAPK cascade (GO:0043410; P=1.88e-2), and positive regulation of response to stimulus (GO:0048584; P=1.53e-2). Unsurprisingly, we also identified a cluster consisting of Toll-like receptor-related cascades, which are well known to be related to monocytes; a similar separate cluster consisting of processes related to immunodeficiency and diseases of the immune system emerged, as well.

### PANDA identifies important markers related to breast cancer

**Extended Data Fig. 5 |:**
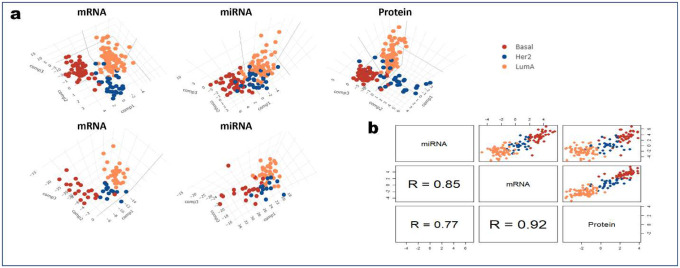
DIABLO results for the multi-omics breast cancer data from TCGA. **a,** 3D scatter plots of DIABLO-extracted components for the training data (first row) and 3D projections of the test data obtained using the transformation matrices learned using DIABLO on the training data (second row). **b,** Pairs plot based on the first component for each omics (mRNA, miRNA, and protein) for the training data. Cross-omics correlations are shown in the lower triangular panels, and scatter plots are shown in the upper triangular panels.

**Fig. 4 |:**
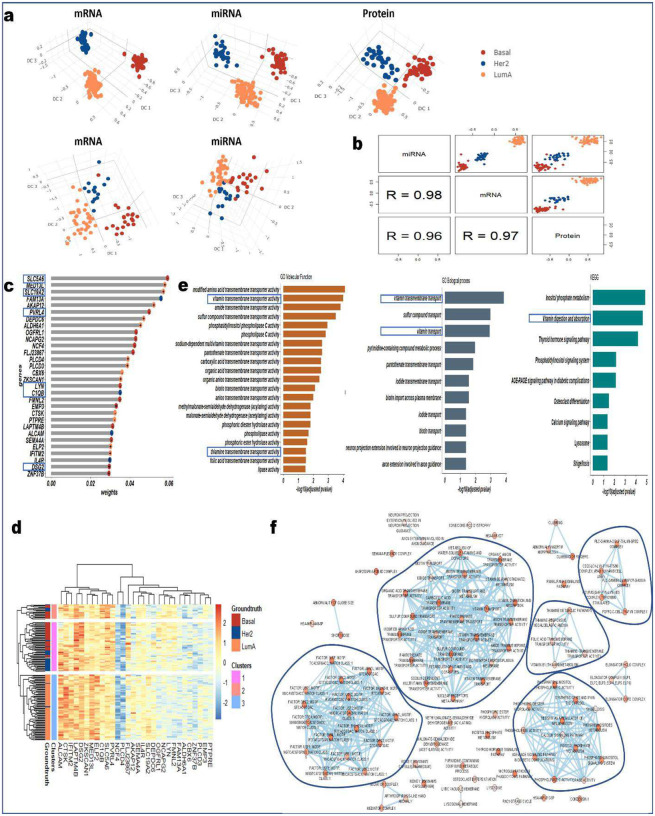
Application to multi-omics breast cancer data from TCGA. **a,** 3D scatter plots of PANDA-extracted components on the training data (first row) and 3D projections of the test data obtained using the transformation matrices learned on the training data (second row). **b,** Pairs plot of the first component for each omics (mRNA, miRNA, and protein) on the training data. Cross-omics correlations are shown in the lower triangular panels, and scatter plots are shown in the upper triangular panels. **c,** Genes with the largest absolute weights (x-axis) on the first mRNA component 1 (DC_1). The colored dots on the right indicate the tumor types from **a** in which the genes had maximum levels of expression. The symbols inside the colored dots indicate the sign of the weights. **d,** Hierarchical clustering heat map based on the top 30 genes (z-scores values) in c. The rows indicate the three breast tumor subtypes, and the columns indicate the top 30 genes with the largest weights on PANDA latent component 1. **e,** Results of gene set enrichment analysis using the top 30 genes in **c.** The false discovery rate-adjusted P-values for the most significantly enriched GO processes and KEGG pathways are shown. **f,** Enrichment maps of the GO processes and KEGG pathways in **d.** Similar pathways are connected using lines, and the line thickness indicates the extent of gene overlap between pathways.

To further highlight the robustness and versatility of the PANDA algorithm, we conducted a series of experiments with The Cancer Genome Atlas (TCGA) breast cancer multi-omics dataset from the mixOmics R package^50^. First, we used the training dataset to learn the PANDA transformation matrix, which we then used to determine lower dimensional representations of both the training and testing sets (Fig. 4a). Worth noting is that, different from the training set, which contains three modalities of data (mRNA, microRNA [miRNA], and protein), the testing set contains only two modalities (mRNA and miRNA). Thus, when determining the latent representations for the mRNA and miRNA modalities in the testing set, we used only the transformation matrix for the mRNA and miRNA modalities learned using the training set. Inspection of the 3D latent representations (Fig. 4a) for both the training and testing demonstrated that PANDA effectively captures the correspondence structures in the different modalities by obtaining common subspaces with similar data patterns while providing accurate grouping of samples in terms of tumor type. Specifically, PANDA obtained discriminant latent representations with similar data structures, indicating shared evidence across the three types of omics data. Quantitatively, this observation is supported by PANDA higher cluster purity values of 1 for the training mRNA, miRNA, and protein latent representations and cluster purity values of 0.93 and 0.81 for the test mRNA and miRNA latent representations. These results are much better than those obtained using DIABLO (Extended Data Fig. 5a), demonstrating cluster purity values of 0.93, 0.85, and 0.91 for the training mRNA, miRNA, and protein latent representations, respectively, and cluster purity values of 0.9 and 0.76 for the test mRNA and miRNA latent representations, respectively. To assess the robustness of the PANDA latent components for classification problems, we concatenated the first 10 latent components for the training mRNA and miRNA omics datasets and used them to train a decision tree (XGBoost) classifier. We then used the corresponding concatenated latent components for the testing mRNA and miRNA datasets to predict the tumor subtypes for the test samples. For cases with more than two modalities of data, a classifier can be built separately using the latent representations obtained for each modality, and the majority voting approach can be used to determine the final classification results for the test samples. In terms of classification accuracy, PANDA resulted in a classification accuracy rate of 87%, compared with 50% for DIABLO.

A good integration algorithm should be able to capture the biological similarities present in the original datasets, thereby maximizing the correlations among latent components of the different omics (cross-omics correlation). To examine whether PANDA latent components capture information from all of the different omics (i.e., maximized cross-omics correlation), we computed the correlation among the first components of the three training omics datasets (Fig. 4b). We observed that PANDA-extracted latent components are highly correlated with each other (indicated by the large correlation coefficient values at the bottom of Fig. 4b), highlighting the ability of PANDA to model the correspondence presenting in the different omics. Also, these latent components maximally separate the different tumor subtypes, further indicating the discriminative power of the components. In comparison, DIABLO-extracted components did well in maximizing the cross-omics correlation but failed to maximize class separation, as samples from different tumor subtypes tended to mix with each other (Extended Data Fig. 5b). Overall, PANDA obtained higher correlation coefficients and had better discriminatory capability than did the DIABLO method.

To gain further insight into the PANDA-extracted latent components and identify important biomarkers related to breast cancer, we investigated the genes, miRNAs, and proteins with the largest absolute weights on the extracted latent components. Even with the small numbers of features in these datasets, PANDA identified and assigned larger weights to the most discriminative genes that best characterize (and/or significantly associate with) the different breast tumor subtypes. For example, latent component 1 explained variations in all tumor subtypes, capturing gene expression signatures strongly associated with the different tumor subtypes (Fig. 4c). A clustering analysis based on only the 30 genes with the largest weights on latent component 1 demonstrated good differential expression patterns, highlighting clusters corresponding to the three breast tumor subtypes (Fig. 4d). Quantitatively, these genes yielded ARI and NMI values of 0.69 and 0.6, respectively.

In addition to capturing discriminant information, the top mRNA gene, miRNA, and protein features identified by PANDA are associated with breast cancer. For example, several GO terms and KEGG pathways (Fig. 4e) related to SLC5A6 and SLC19A2 (members of the solute carrier family) were significantly enriched, including vitamin transport (GO:0051180; P=1.23e-4), vitamin transmembrane transporter activity (GO:0090482; P=4.82e-5), thiamine transmembrane transporter activity (GO:0015234; P=1.90e-3), and vitamin digestion and absorption (KEGG:04977; P=1.66e-5). Nutrition plays an important role in cancer patients by reducing the risk of mortality and morbidity and accelerating the recovery process. Several studies have demonstrated significant association between vitamin intake and transportation in patients with breast cancer^51–53^. For example, Campbell et al.^54^ showed that a decreased level of vitamin C in breast tumors is associated with severe breast cancer and advanced stages of necrosis. Also, dietary administration of vitamin C has been significantly associated with reduced risk of total (all-cause) and breast cancer-specific mortality^51,55^. Furthermore, vitamin C is reported to inhibit triple-negative breast cancer metastasis and induce apoptosis^56^, making it a good candidate for cancer treatment^57^. Tissue cellular vitamin C levels may be related to expression of and polymorphisms in the genes that encode vitamin C transport; variations in these transporter genes may regulate the active transport of vitamin C and lead to cancer development^52,58^.

Authors reported similar associations for the negatively weighted genes (on PANDA latent component 1) PVRL4 (Nectin-4) and LYN, which are both maximally expressed in basal breast tumors (as indicated by the red color in Fig. 4c). The SRC kinase family member LYN is highly expressed in triple-negative/basal-like breast cancer^59^ and is reported to be a potential drug target for basal-like breast cancer^60^. PVRL4 is mainly expressed during embryogenesis and reported to be consistently expressed in basal tumors^61,62^. Athanassiadou et al.^63^ showed that positive PVRL4 (Nectin-4) expression was strongly associated with increased tumor size, increased tumor grade (II and III), increased lymph node infiltration, and shortened survival. Their findings also suggest that overexpression of PVRL4 is a good marker for aggressive clinical behavior and worst prognosis in breast carcinoma.

Moreover, DSG2 and C1QB were among the genes with the largest weights on PANDA latent component 1. DSG2 is an important cell-cell adhesion molecule associated with poor prognosis and tumor growth in breast cancer patients^64^. Decreased expression of DSG2 enables tumor cells to undergo epithelial-mesenchymal transition, whereas upregulation of DSG2 promotes the formation of circulating tumor cells^64,65^. Chang et al.^65^ reported a significantly (P=0.008) lower distant metastasis-free survival rate in breast cancer patients with high expression of DSG2, indicating that DSG2 plays a key role in breast cancer metastasis and can be considered a prognostic marker. For the C1QB gene which has the maximum expression level in the Her2-positive tumor subtype and was among the genes with the largest weights on PANDA latent component 1, Mangogna et al.^66^ showed that C1QB expression was positively associated with overall survival rate in Her2-positive subtype breast cancer. Similarly, Tsao et al.^67^ assessed the relationship between C1QB expression and overall survival rate for Her2-positive, luminal A, luminal B, and basal (triple-negative) breast cancer patients and found that the expression was associated with a better overall survival rate in patients with Her2-positive breast cancer than in those with other subtypes. These findings demonstrated that C1QB may serve as an important biomarker for Her2-targeted therapy. On the other hand, several GO and KEGG terms are significantly enriched for top genes identified using the DIABLO method, including the AMPK signaling pathway (KEGG:04152; P=3.490e-2), lipid transport (GO:0006869; P=3.43e-2), and cellular response to hypoxia (GO:0071456; P=3.92e-2). Also, DIABLO identified FUT8 and PREX1 as key gene markers.

An enrichment map of the significantly enriched biological processes revealed five clusters, with the largest one comprising of pathways involved in vitamin transporter activities (Fig. 4f). Thiamine (vitamin B1)-related pathways, including thiamine metabolic pathways (WP:WP4297; P=2.98e-2), thiamine transmembrane transporter activity (GO:0015234; P=1.41e-2), and vitamin B1 metabolism (REAC:R-HSA-196819; P=1.82e-2), were also significantly enriched and formed a separate small cluster. Furthermore, phospholipase-related pathways as well as resistin as a regulator of inflammation (WP:WP4481; P=1.34e-3) and the AGE-RAGE signaling pathway involved in diabetic complications (KEGG:04933; P=6.77e-3) were enriched and grouped in a large cluster.

### PANDA-extracted components are robust predictors of clinical outcome

**Extended Data Fig. 6 |:**
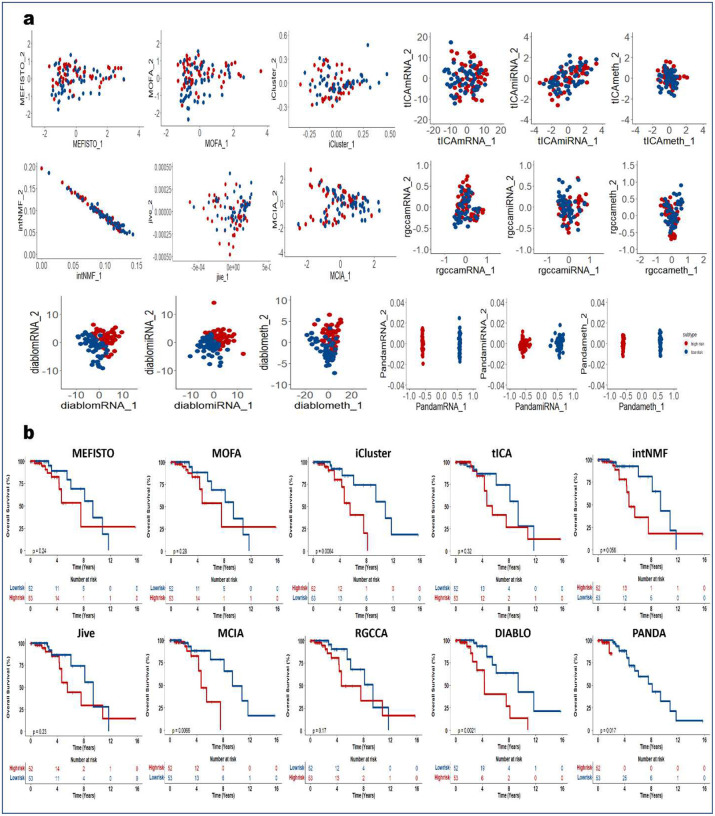
Benchmarking PANDA with SOTA multi-omics integration methods for clinical outcome prediction using the multi-omics breast cancer data from TCGA. **a,** Scatter plots of the first two extracted components for the different multi-omics integration methods. Each dot corresponds to a sample (patient), and the colors red and blue denote high- and low-risk patients, respectively. **b,** Kaplan-Meier plots obtained by splitting the samples into two groups based on the first extracted component for each method. The optimal cut points for the variables were determined using the maximally selected rank statistics approach.

**Extended Data Fig. 7 |:**
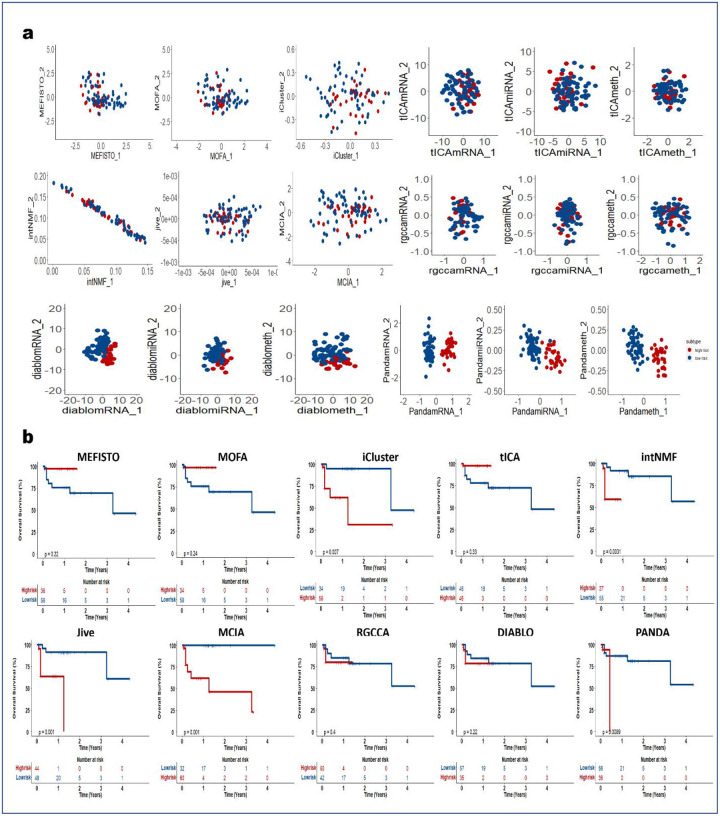
Benchmarking PANDA with SOTA multi-omics integration methods for clinical outcome prediction using the multi-omics colon cancer data from TCGA. **a,** Scatter plots of the first two extracted components for the different multi-omics methods. Each dot corresponds to a sample (patient), and the colors red and blue denote high- and low-risk patients, respectively. **b,** Kaplan-Meier plots obtained by splitting the samples into two groups based on the first extracted component for each method. The optimal cut points for the variables were determined using the maximally selected rank statistics approach.

**Extended Data Fig. 8 |:**
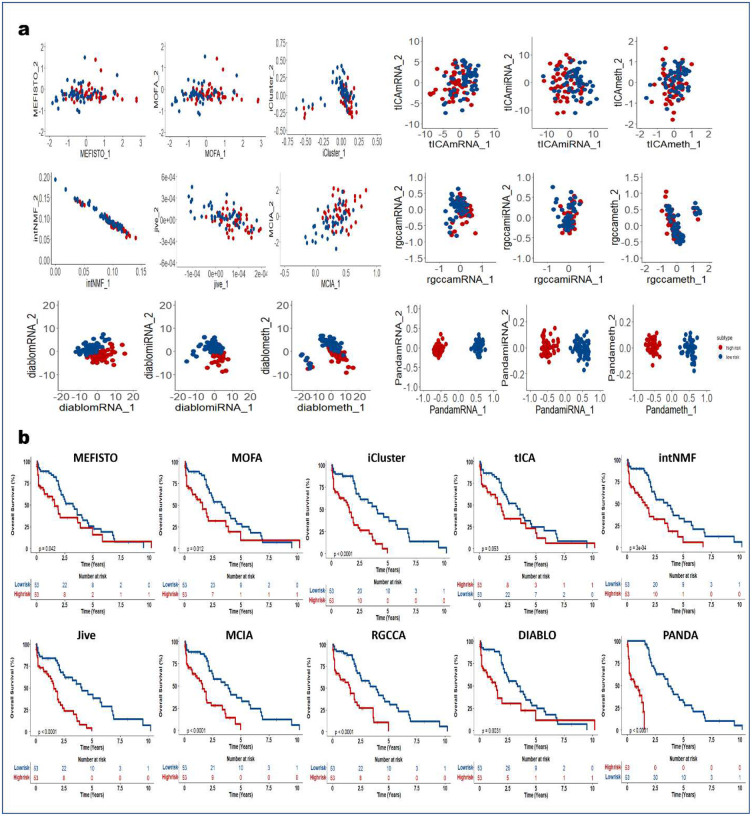
Benchmarking PANDA with SOTA multi-omics integration methods for clinical outcome prediction using the multi-omics lung cancer data from TCGA. **a,** Scatter plots of the first two extracted components for the different multi-omics methods. Each dot corresponds to a sample (patient), and the colors red and blue denote high- and low-risk patients, respectively. **b,** Kaplan-Meier plots obtained by splitting the samples into two groups based on the first extracted component for each method. The optimal cut points for the variables were determined using the maximally selected rank statistics approach.

**Extended Data Fig. 9 |:**
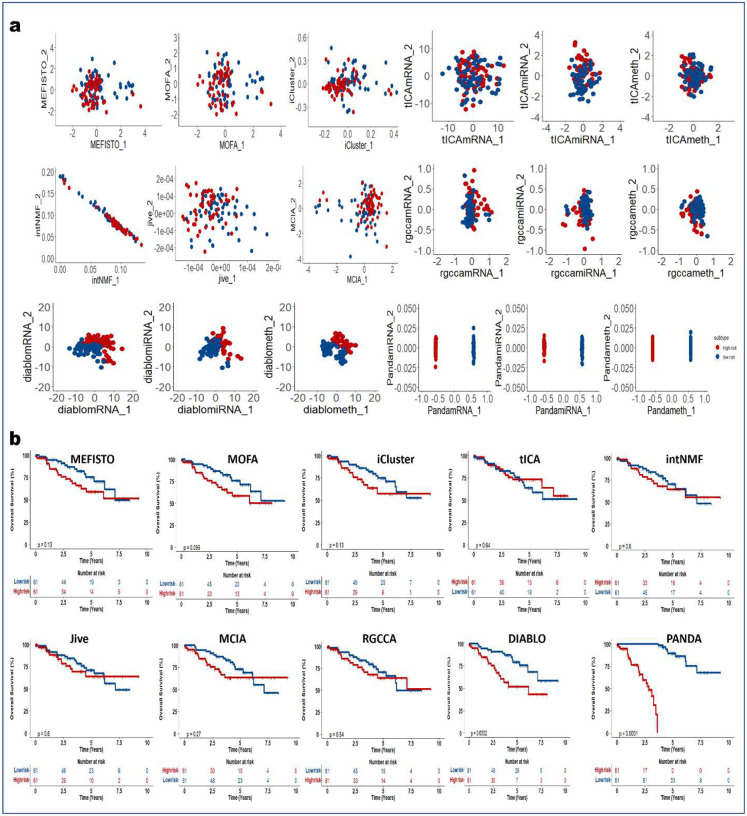
Benchmarking PANDA with SOTA multi-omics integration methods for clinical outcome prediction using the multi-omics kidney cancer data from TCGA. **a,** Scatter plots of the first two extracted components for the different multi-omics methods. Each dot corresponds to a sample (patient), and the colors red and blue denote high- and low-risk patients, respectively. **b,** Kaplan-Meier plots obtained by splitting the samples into two groups based on the first extracted component for each method. The optimal cut points for the variables were determined using the maximally selected rank statistics approach.

**Extended Data Fig. 10 |:**
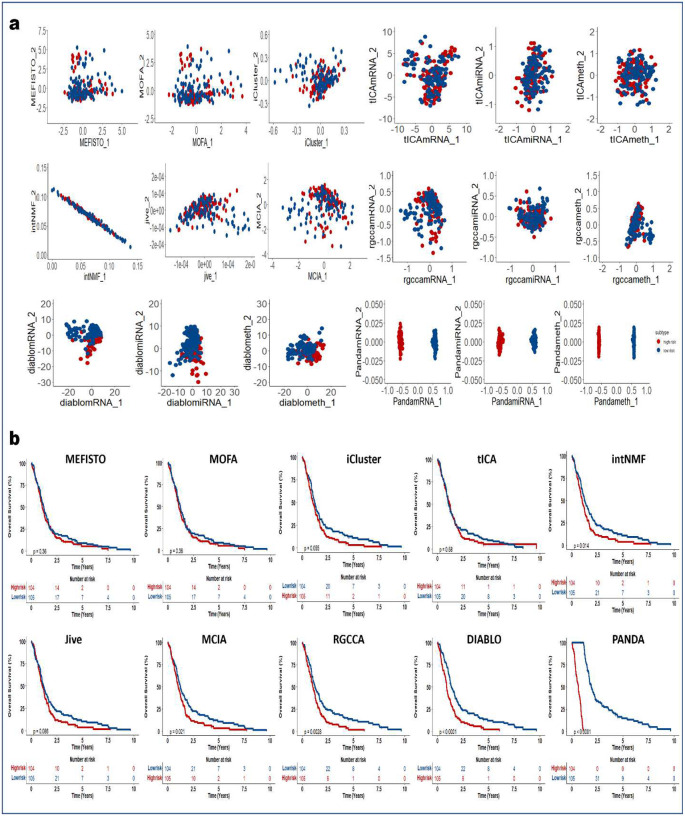
Benchmarking PANDA with SOTA multi-omics integration methods for clinical outcome prediction using the multi-omics GBM data from TCGA. **a,** Scatter plots of the first two extracted components for the different multi-omics methods. Each dot corresponds to a sample (patient), and the colors red and blue denote high- and low-risk patients, respectively. **b,** Kaplan-Meier plots obtained by splitting the samples into two groups based on the first extracted component for each method. The optimal cut points for the variables were determined using the maximally selected rank statistics approach.

**Fig. 5 |:**
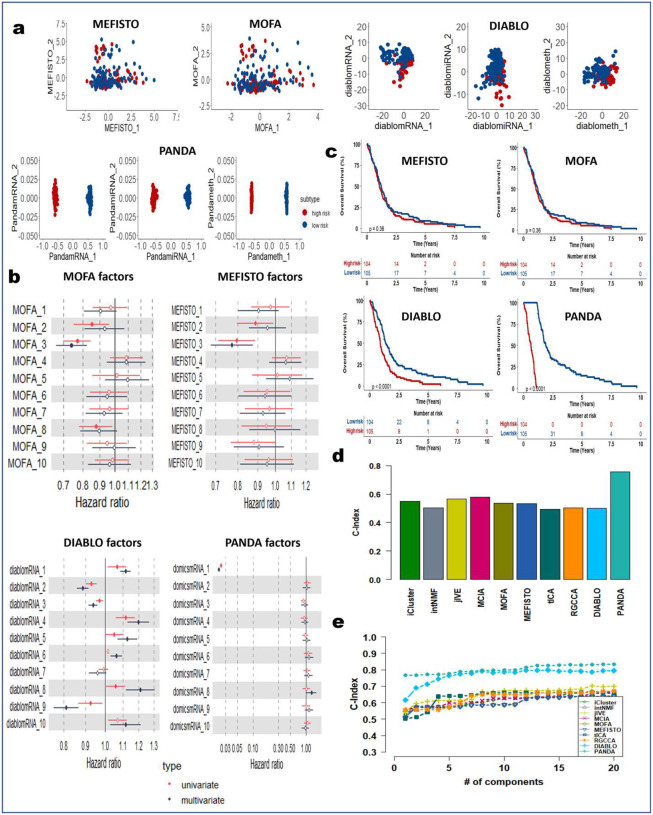
Benchmarking PANDA with SOTA multi-omics data integration methods for clinical outcome prediction using multi-omics GBM data from TCGA. **a,** Scatter plots of the first two extracted components for the different multi-omics methods. Each dot corresponds to a sample (patient), and the colors red and blue denote high- and low-risk patients, respectively. **b,** Results of univariate (red) and multivariate (black) Cox proportional hazards models based on components extracted using the different multi-omics methods. **c,** Kaplan-Meier plots obtained by splitting the samples into two groups based on the first extracted component of each method. The optimal cut points for the variables were determined using the maximally selected rank statistics approach. **d,** Bar plot of the performance (c-index) of the Cox proportional hazards models using clustering labels obtained by applying hierarchical clustering approach on the 2D projections in **a. e,** Performance results for the Cox proportional hazards models with different numbers of extracted components using the different multi-omics methods. Of note is that for methods that can extract separate components for the different omics (mRNA, miRNA, and methylation), the mRNA extracted components were used in the Cox proportional hazards models.

We next demonstrated how PANDA latent representations can be used as predictors in models of clinical outcomes. We applied the PANDA method to five TCGA cancer multi-omics datasets (Table 1) and compared its performance with the different SOTA multi-omics integration methods described in the previous sections. We adopted an approach similar to that employed in the DIABLO method paper^4^ to split patients into low-and high-risk groups. We then extracted latent representations of the datasets and used the ARI and NMI metrics to quantitatively evaluate the clustering results based on the latent representations of the different compared integrative multi-omics methods. Consensus clustering of the first two PANDA latent representations (Fig. 5a, Extended Data Figs. 6a, 7a, 8a, 9a, and 10a) of the five different datasets clearly stratified the patients into two groups (low- and high-risk), yielding both ARI and NMI values of 1. However, all of the compared approaches produced very low ARI and NMI values close to zero as evidenced by considerable overlap of the two groups in the obtained 2D latent spaces. The DIABLO method performed better than the rest of the compared approaches but still failed to recover the group patterns based on only its first two components, requiring more components to provide satisfactory group separations.

Next, we examined the association of the extracted PANDA latent components with overall survival, using a Cox proportional hazards regression model. We investigated these associations under both univariate and multivariate settings. Of note, we used the mRNA latent representations for methods that seek omics-specific representations. PANDA latent component 1 on the glioblastoma multiforme (GBM) datasets was markedly associated with overall survival time in both the univariate and multivariate analysis, whereas PANDA latent component 8 was markedly associated with that in the multivariate analysis alone (Fig. 5b). PANDA latent component 1 was related to disease progression, as this latent component alone is able to perfectly separate the low- and high-risk patients (Fig. 5a) by assigning strictly negative and positive component values for high- and low-risk patients, respectively. Similarly, some of the latent components extracted using the MOFA, MEFISTO and DIABLO approaches were markedly associated with overall survival. Furthermore, we plotted Kaplan-Meier survival curves based on the first latent components of the different approaches (Fig. 5c, Extended Data Figs. 6b, 7b, 8b, 9b, and 10b). We observed that PANDA provides perfect stratification of the patients with the low- and high-risk curves diverging very early and with log-rank test statistics that were very significant for all five TCGA datasets. On the other hand, a majority of the compared approaches, including MOFA and MEFISTO, could not determine latent components that provided good stratification of the patients. MCIA and DIABLO achieved the best performance among all of the benchmarked approaches, as they determined factors significantly associated with overall survival in four of the five datasets (Extended Data Figs. 6b, 7b, 8b, 9b, and 10b).

**Extended Data Fig. 11 |:**
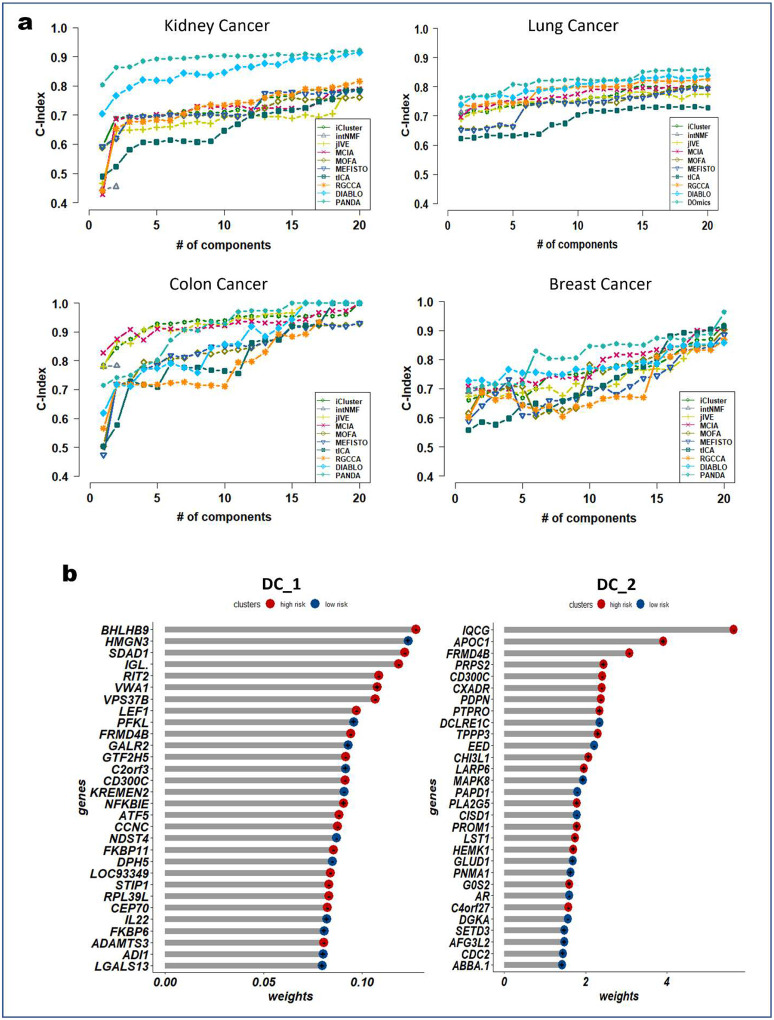
Additional results from benchmarking PANDA with SOTA multi-omics integration methods on the TCGA datasets. **a,** Performance results of Cox proportional hazards models with different numbers of extracted components for the different multi-omics methods. Of note is that for methods that can extract separate components for the different omics (mRNA, miRNA, and methylation), the mRNA extracted components were used in the Cox proportional hazards models. b, Genes with the largest absolute weights (x-axis) on mRNA latent components 1 and 2. The colored dots on the right indicate the risk group (high- vs. low-risk) in which the genes had maximum expression. The symbols inside the colored dots indicate the sign of the weights.

We also examined the association between overall survival and the clustering results based on the lower dimensional latent representations for the different omics approaches (Fig. 5a, Extended Data Figs. 6a, 7a, 8a, 9a, and 10a). Specifically, we used the clustering results as covariates in a univariate Cox proportional hazards model and investigated their predictive power in terms of concordance index. The PANDA model yielded a much higher concordance index for the GBM dataset than did the compared approaches (Fig. 5d). In addition, we investigated the predictive performance when iteratively varying the number of latent components (from 1 to 20) in the Cox proportional hazards model. PANDA consistently outperformed the other methods when varying the number of latent components for the GBM dataset (Fig. 5e). We observed similar good performance of the PANDA method with the remaining datasets, achieving a concordance index value of 1 for the colon and breast cancer datasets (Extended Data Fig. 11a). The DIABLO method also performed well in this analysis, most especially with the GBM and kidney cancer datasets, for which PANDA and DIABLO both outperformed the other methods by large margins for all of the varying number of latent components. This further highlights the predictive power of the discriminant analysis-based approaches. Overall, PANDA latent components are robust and significantly associated with patient’s overall survival.

In addition, we inspected the PANDA latent components to identify marker genes that associated with biological processes and pathways. Similar to our biomarker identification analysis described above, we identified the genes in the GBM dataset with the largest weights on the PANDA latent components (Extended Data Fig. 11b). Several of these genes were previously associated with GBM. For example, RIT2, which belongs to the Ras signaling gene family and is involved in neuronal development, was significantly enriched on PANDA latent component 1. The Ras signaling pathway is hyperactive in malignant gliomas and has a broad impact on oncogenesis and tumor biology, making it an important therapeutic target for malignant gliomas^68^. Also, NFKBIE, which has maximum expression in the high-risk groups along PANDA latent component 1, is reported to associate with GBM progression. Friedmann-Morvinski et al.^69^ showed that inhibition of NFKBIE in human GBMs is strongly associated with reduced cell proliferation and impaired tumor progression, making NFKBIE an attractive therapeutic target for GBM.

Apolipoprotein C1 (APOC1) which has a larger weight on PANDA latent component 2, is reported to promote tumorigenesis and progression of many types of cancer^70,71^. Zheng et al.^71^ showed that APOC1 was highly expressed in GBM tissues and that its expression was associated with disease progression, which is in line with the high expression of APOC1 seen in the high-risk group along PANDA latent component 2. In general, APOC1 plays a pivotal role in GBM tumorigenesis, causing resistance to ferroptosis, and may be a promising therapeutic target for GBM^71^.

The mitogen activated protein kinase 8 (MAPK8) has a larger weight on PANDA latent component 2 and was highly expressed in the low-risk group. Consistent with our observation, high expression of MAPK8 is reported to be significantly associated with favorable outcome (lengthened survival)^72,73^. However, overexpression of MAPK8 is also reported to be associated with the MAPK signaling pathway activated in temozolomide-resistant glioblastoma cells^74^. Specifically, MAPK8 promoted resistance of GBM cells to temozolomide, accelerated cell proliferation, and induced apoptosis of GBM cells by upregulating the MAPK signaling pathway.

### PANDA has shorter runtimes than do DIABLO, MEFISTO, and MOFA

To analyze the computational time requirement of the PANDA method in comparison with those for the DIABLO, MEFISTO, and MOFA methods, we benchmarked the runtime requirements of these methods using the PBMC datasets. We carried out all experiments using a Dell desktop computer equipped with an Intel Xeon Silver 4210 processor and 256 GB of memory. Initially, we analyzed the time taken for each method to extract 100 latent components, observing the best performance (minimal runtime) with the PANDA method (≈9.64 minutes) followed by the DIABLO method (≈15.86 minutes). The MOFA algorithm took about 1.19 hours to extract the 100 latent components, whereas MEFISTO failed to extract the 100 components and ran into a memory issue (could not allocate ≈80GB of memory). Thus, we reduced the number of components to 50, and after running the MEFISTO algorithm for more than 24 hours without returning any results, we terminated the process. Overall, we found PANDA to be faster than the DIABLO, MOFA, and MEFISTO algorithms, making it suitable for use with large-scale datasets.

### Extensive ablation studies and parameter sensitivity analysis further demonstrate the robustness of PANDA

#### Ablation study

**Extended Data Fig. 12 |:**
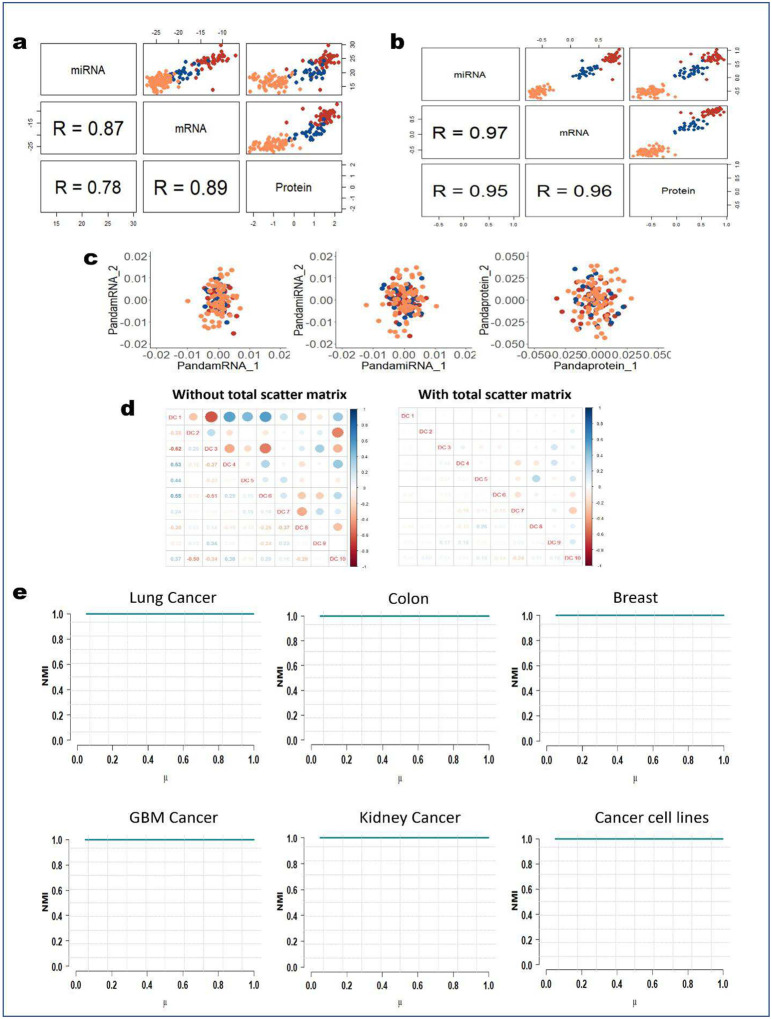
Results of an ablation study performed to highlight the importance of the different terms (parts) of the proposed PANDA objective function. **a,** Pairs plot obtained by considering only the between-class scatter term in the PANDA objective function. **b,** Pairs plot obtained by considering both the between-class and total scatter terms in the PANDA objective function. **c,** Scatter plots obtained by considering only the consistency and total scatter terms in the PANDA objective function. **d,** The correlation structures for the top 10 mRNA components extracted by solving the PANDA objective function with and without the uncorrelated constraint (total scatter matrix). **e,** The clustering performance in terms of NMI score (y-axis) for the first two mRNA extracted components with different values of μ (x-axis) was examined.

We conducted several experiments to validate the contribution of each term in the PANDA objective function (Eq. 15 in the Methods section). As evident from the objective function, PANDA consists of three terms: 1) the between-class scatter term $\left(W^{T}BW\right)$, 2) the correspondence/consistency term ($W^{T}MW$), and 3) the total scatter term $\left(W^{T}CW\right)$, which encompasses the uncorrelated constraint. We constructed several disintegrated PANDA models by removing some terms from each model and examined the performance of the disintegrated models with the TCGA breast cancer multi-omics datasets from the mixOmics package. Because the between-class scatter term is the basis for our PANDA model, we initially considered it to be the only term in the objective function and examined the performance of the disintegrated PANDA model. The between-class scatter term captures discriminant structures in the data, but we observed little overlap among samples of the three breast cancer subtypes (Extended Data Fig. 12a). At the same time, the correlations among the latent representations of the different omics were maximized using only this term. However, when we combined the between-class scatter term with the total scatter term, we observed great improvement in terms of class (breast cancer subtype) separation as well as maximizing the correlation among the latent representations of the different omics (Extended Data Fig. 12b).

Next, we examined the performance of PANDA considering only the correspondence and total scatter terms in the objective function. This combination enabled PANDA to obtain latent representations with similar data structures but failed to capture enough discriminative information to separate the different cancer subtypes (Extended Data Fig. 12c). Thus, the between-class and total scatter terms promoted each other and jointly worked in collaboration to optimally enhance the discriminant performance of PANDA.

Lastly, we examined how the total scatter term helps extract uncorrelated latent components. Specifically, we examined the correlations among the extracted latent components based on the disintegrated PANDA model without the total scatter term and those extracted based on the complete (with total scatter term) PANDA model (Extended Data Fig. 12d). The total scatter term is very crucial to the PANDA model, as it significantly decreases the level of correlation among the PANDA latent components. As evident from the correlation matrices, there exist relatively high correlation among some of the extracted latent components based on the disintegrated PANDA model (without the total scatter term), whereas the extracted latent components based on the complete PANDA model (with the total scatter term) were very independent and uncorrelated. In summary, all three terms in the PANDA objective function play important and indispensable roles, as they work together to provide the best performance of the PANDA model.

#### Parameter sensitivity and computational complexity

The proposed PANDA algorithm has only one hyperparameter: the regularization parameter (η). The choice of this parameter is important to the performance of the PANDA algorithm and depends on the structure of the data. We randomly chose this hyperparameter in our experiments. Here, we examined the sensitivity of the PANDA algorithm with respect to varying values of the hyperparameter η. Specifically, we chose the hyperparameter values from among a wide range of values η∈(0,1] and extracted PANDA latent representations for the cancer cell line and the five TCGA cancer datasets. We then assessed the effectiveness of the latent representations in capturing the discriminant structure in the data under different values of η using the NMI metric (Extended Data Fig. 12e). We observed that the PANDA algorithm was very stable (using all the six datasets) with respect to hyperparameter η. The choice of this hyperparameter did not affect the performance of PANDA, as it consistently achieved good performance for values of η∈(0,1] with all six datasets. In general, PANDA was robust and less sensitive to the choice of the hyperparameter η. Next, we analyzed the computational complexity of the PANDA algorithm. The most time-consuming operation using this algorithm is solving the generalized eigenproblem in Eq. 19 in the Methods section. The computational complexity of computing the generalized eigenproblem is $O\left(m^{3}\right)$. Thus, the PANDA algorithm scales well in practice.

## Discussion

PANDA approaches multi-omics data integration as a discriminant feature extraction problem by determining common discriminant latent spaces for the various omics data. Recently, the number of annotated multi-omics datasets produced using advanced technologies has increased greatly. PANDA takes advantage of these annotated datasets to better predict important clinical factors or outcomes such as cancer subtype and patient overall survival. As with other multi-omics integration methods, PANDA extracts latent representations that capture major sources of biological variations that may arise owing to sample heterogeneity. A major distinction of PANDA from other SOTA multi-omics integration methods is that it explicitly models multi-omics correspondence (Table 2) by finding common discriminant latent spaces that share similar data structures. Other integrative multi-omics methods, such as DIABLO, MEFISTO, and intNMF, can be easily affected by technological variations (Table 2) and the presence of confounding features^4^, as they are not designed to handle these problems. Owing to the correspondence modeling strategy, PANDA can overcome this problem through minimizing the differences in the distribution of individual omics datasets in a common discriminant latent space. The ability of PANDA to explicitly model multi-omics correspondence is especially useful, since strong biological relationships exist among these omics datasets and these high-dimensional observations are irregularly sampled from different groups. Additionally, PANDA adds immense value in cases in which the omics datasets are highly noisy and heterogeneous. PANDA determines omics-specific latent representations that we demonstrated can enable denoised representations of the datasets and improve the accuracy of discriminant analysis. Using a simulated single-cell multi-omics dataset with high levels of noise and sparsity, we demonstrated that PANDA achieved substantially better performance in terms of denoised representations and determined better discriminant latent spaces than did the benchmarked integrative multi-omics methods. Also, using real-world datasets, including single-cell and TCGA multi-omics datasets, we showed that integrative analysis using PANDA facilitates the identification of important biomarkers that are strongly associated with disease outcomes (clinical behavior) and linked to reported promising therapeutic targets. Our analysis using the five TCGA cancer multi-omics datasets demonstrated that PANDA latent components are robust predictors of patient clinical outcome and provide significantly better grouping of patients (low- vs. high-risk) than do the benchmarked methods.

In addition to rich discrimination and characterization of samples, PANDA can uncover correlation structures such as the relationship between RNA and protein molecules within a cell. The use of multi-omics correspondence in conjunction with multi-omics correlations is more efficient than the use of multi-omics correlations alone (as implemented in RGCCA and DIABLO). We observe that the magnitude of cross-omics correlations among the PANDA latent components is much greater than that of the raw correlations among pairs of the original omics features, which may be a result of high levels of noise in the original datasets. Compared with other discriminant analysis methods, such as DIABLO, PANDA latent components are very discriminative and captures cross-omics correlations. DIABLO and other related approaches must determine a compromise or trade-off between discrimination and correlations, which requires prior domain knowledge (omics known biological associations)^4^. This presents a large challenge when identifying biologically meaningful biomarkers. In contrast, PANDA does not require prior domain knowledge and can effectively model (without any compromise) both the discriminant and correlation structures, overcoming the limitations of the discriminant-based multi-omics integration methods.

The choice of regularization parameter in PANDA is essential to the optimal performance of the method. We found that consistently choosing the value of this parameter within the range (0,1] provides stable, optimal results. Similar to other multi-omics integration methods, the number of PANDA latent components can be considered tunable, as well, although PANDA has good performance even with a small number of latent components.

The superior performance and robustness of PANDA when compared with the benchmarked multi-omics methods in our analysis demonstrate the effectiveness of PANDA as a useful tool for researchers interested in discriminant and integrative analysis of multi-omics data to reveal biological processes driving sample heterogeneity and the relationships among different omics data. Furthermore, PANDA simplifies discriminant and integrative analysis of multi-omics data through a single pipeline that jointly analyzes different omics datasets; otherwise, this analysis would have been performed through separate pipelines, in which the different results must be combined afterward in an inefficient way. The joint discriminant analysis with correspondence structure modeling greatly enhances the ability of PANDA to minimize technical variations and preserve important biological signals.

Although we focused on the application of PANDA to bulk, and single-cell transcriptomics data, the model readily extends to accommodate more, or different modalities of data provided the data measurements are on a continuous scale. Our benchmarking results highlight the versatility of PANDA with different types of multi-omics datasets, as it consistently achieved the best performance across all of the benchmarking analyses.

Like other multi-omics integration methods, PANDA has its limitations. First, the method is linear and may not perform well when the datasets are highly nonlinear. Second, PANDA was developed primarily for data with continuous variables; we have not explored how it performs with other types of datasets, such as discrete and categorical datasets. A promising future direction will be to extend PANDA to handling other data types and cases in which the datasets are highly nonlinear.

In summary, we developed a novel method for the joint discriminant and integrative analysis of multi-omics datasets. PANDA can extract salient and rich discriminative latent representations, providing joint visualizations that are robust to technical variations while capturing important biological variations. The concept of explicitly modeling cross-omics correspondence as implemented in PANDA can also be adapted to other integrative analysis methods, such as DIABLO and MEFISTO, to tackle the technical variation issue affecting these methods.

## Methods

### PANDA algorithm

In this study, we proposed a novel multi-omics joint discriminant analysis algorithm PANDA, which effectively finds linear transformations for each omics to map samples from different omics into discriminant common latent spaces. PANDA determines discriminant common latent spaces by maximizing the multi-omics between-class variation and minimizing the multi-omics within-class variation computed using examples across all omics. This means samples belonging to different classes are mapped far apart from each other and samples belonging to the same class are mapped close to each other in the discriminant common latent spaces. In addition, PANDA captures cross-omics correlation and consistency while ensuring that the extracted latent components of each omics (omics-specific components) are not redundant (i.e., not highly correlated with each other). As inputs, PANDA expect a collection of V omics matrices $X_{v}\in R^{m_{v}\times n}$,v=1,…,V, where $m_{v}$ denotes the number of features (e.g., mRNA, miRNA, methylation) in omics v,n denotes the number of samples, and we denote by c=1,…,C the number of classes/subtypes. To maximize between-class variations in the discriminant common latent spaces, we consider maximizing the total covariance of the different omics:

(1) $$\underset{w_{1},\ldots ,w_{V}}{\text{argma}x\;}\sum_{v=1}^{V}{\left[cov\left(X_{v}w_{v},Y\right)\right]}^{2},$$

where $w_{v}\in R^{p}$ is a projection vector of the vth omics and Y denotes the class membership matrix

(2) $$Y=\left(\begin{array}{cccc} 1_{n_{1}} & 0_{n_{1}} & \cdots & 0_{n_{C}}\\ 0_{n_{2}} & 1_{n_{2}} & \cdots & 0_{n_{C}}\\ \vdots & \vdots & \ddots & \vdots \\ 0_{n_{C}} & 0_{n_{C}} & \cdots & 1_{n_{C}} \end{array}\right),$$

where $n_{i}$ (for i=1,…,C) represents the number of samples in the ith class, $\sum_{i=1}^{C}n_{i}=n$ (total number of samples), and $0_{n_{i}}$ and $1_{n_{i}}$ are $n_{i}\times 1$ vectors of zeros and ones, respectively. Using simple algebra, we can show that maximizing the criterion in Eq. 1 is the same as maximizing the total between-class variation of the different omics.

Since there exists a huge amount of correspondence between the different omics. For example, in transcriptomics, a sequence of a gene’s DNA is copied (transcribed) to make an RNA molecule. A good multi-omics data integration algorithm should be able to capture such kind of relationships. Thus, the correspondence between the different omics was modeled by minimizing

(3) $$\sum_{v,k=1}^{V}{\left\|\psi_{v}-\psi_{k}\right\|}_{2}^{2},$$

where $\psi_{v}={\left(X_{V}^{T}X_{v}\right)}^{-1}X_{v}^{T}w_{v}$ is the least-squares representation to the formulation (from representer theorem^75^)

(4) $$w_{v}=X_{v}\psi_{v}$$

and $\psi_{v}$ captures the structure of $w_{v}$.

Minimizing the criterion (Eq. 3) will enable not only capture of the common latent structures shared by all omics but also exploitation of the complementary information within the different omics, which is very important in multi-omics data analysis. Combining Eq. 1 and Eq. 3, the objective function of PANDA can be written as

(5) $$\underset{w_{1},\ldots ,w_{V}}{\text{argma}x\;}\sum_{v=1}^{V}{\left[cov\left(X_{v}w_{v},Y\right)\right]}^{2}-\eta \sum_{v,k=1}^{V}{\left\|\psi_{v}-\psi_{k}\right\|}_{2}^{2},$$

where η≥0 is the regularization parameter. The first term in Eq. 5 can be reformulated as follows:

(6) $$\sum_{v=1}^{V}{\left[cov\left(X_{v}w_{v},Y\right)\right]}^{2}=\sum_{v=1}^{V}\frac{1}{{\left(n-1\right)}^{2}}w_{v}^{T}X_{v}^{T}HYY^{T}HX_{v}w_{v}\;=\frac{1}{{\left(n-1\right)}^{2}}\left(n_{1}\left(\mu_{1}-\mu \right)n_{2}\left(\mu_{2}-\mu \right)\ldots n_{C}\left(\mu_{C}-\mu \right)\right)\left(\begin{array}{c} n_{1}\left(\mu_{1}-\mu \right)\\ n_{2}\left(\mu_{2}-\mu \right)\\ \vdots \\ n_{C}\left(\mu_{C}-\mu \right) \end{array}\right)\;=\frac{1}{{\left(n-1\right)}^{2}}\sum_{i=1}^{C}n_{i}^{2}\left(\mu_{1}-\mu \right){\left(\mu_{1}-\mu \right)}^{T}\;=\frac{1}{{\left(n-1\right)}^{2}}\sum_{i=1}^{C}n_{i}^{2}\left(\mu_{i}\mu_{i}^{T}-\mu_{i}\mu^{T}-\mu \mu_{i}^{T}+\mu \mu^{T}\right)\;=\frac{1}{{\left(n-1\right)}^{2}}\left(\sum_{i=1}^{C}\;n_{i}^{2}\mu_{i}\mu_{i}^{T}-\xi \mu \mu^{T}\right)\;=\frac{1}{{\left(n-1\right)}^{2}}(\sum_{i=1}^{C}\;n_{i}^{2}\left(\frac{1}{n_{i}}\sum_{r=1}^{V}\;n_{ir}w_{r}^{T}{\left(\mu_{ir}^{\left(x\right)}\right)}^{T}\right)\left(\frac{1}{n_{i}}\sum_{j=1}^{V}\;n_{ij}\mu_{ij}^{\left(x\right)}w_{j}\right)-\xi \left(\frac{1}{n}\sum_{i=1}^{C}\;\sum_{r=1}^{V}\;n_{ir}w_{r}^{T}{\left(\mu_{ir}^{\left(x\right)}\right)}^{T}\right)\left(\frac{1}{n}\sum_{i=1}^{C}\;\sum_{j=1}^{V}\;n_{ij}w_{r}^{T}\mu_{ij}^{\left(x\right)}w_{j}\right))\;=\sum_{r=1}^{V}\sum_{j=1}^{V}w_{r}^{T}\left[\sum_{i=1}^{C}\frac{n_{ir}n_{ij}}{{\left(n-1\right)}^{2}}{\left(\mu_{ir}^{\left(x\right)}\right)}^{T}\mu_{ij}^{\left(x\right)}-\delta \left(\sum_{i=1}^{C}\;n_{ir}{\left(\mu_{ir}^{\left(x\right)}\right)}^{T}\right)\left(\sum_{i=1}^{C}\;n_{ij}\mu_{ij}^{\left(x\right)}\right)\right]w_{j}\;=\sum_{r=1}^{V}\sum_{j=1}^{V}w_{r}^{T}B_{rj}w_{j}\;=W^{T}BW.$$

where $H=I_{n}-\left(\frac{1}{n}\right)1_{n}1_{n}^{T}$ is the usual centering matrix ($I_{n}$ is the n×n identity matrix), $\mu_{i}=\frac{1}{n_{i}}\sum_{r=1}^{V}\sum_{k=1}^{n_{ir}}x_{ik}^{\left(r\right)}w_{r}$ is the mean of all samples of the ith class over all omics in the shared space, $\mu =\frac{1}{n}\sum_{i=1}^{C}\sum_{r=1}^{V}\sum_{k=1}^{n_{ir}}x_{ik}^{\left(r\right)}w_{r}$ is the mean of all samples over all omics in the shared space, $\mu_{ir}^{\left(x\right)}=\frac{1}{n_{ir}}\sum_{k=1}^{n_{ir}}x_{ik}^{\left(r\right)}$ is the mean of all samples of the ith class from the rth omics in the original space, $n_{i}=\sum_{r=1}^{V}n_{ir}$ is the number of samples in the ith class in all omics, $n=\sum_{i=1}^{C}n_{i}$ is the number of samples from all classes in all omics, $n_{ir}$ is the number of samples in the ith class of the rth omics, $\delta =\frac{\xi }{(n-1{)}^{2}}$, $\xi =\sum_{i=1}^{C}n_{i}^{2}$ and

(7) $$B_{jr}=\sum_{i=1}^{C}\frac{n_{ir}n_{ij}}{{\left(n-1\right)}^{2}}{\left(\mu_{ir}^{\left(x\right)}\right)}^{T}\mu_{ij}^{\left(x\right)}-\delta \left(\sum_{i=1}^{C}n_{ir}{\left(\mu_{ir}^{\left(x\right)}\right)}^{T}\right)\left(\sum_{i=1}^{C}n_{ij}\mu_{ij}^{\left(x\right)}\right).$$

The multi-omics between-class scatter matrix B is defined as

(8) $$B=\left(\begin{array}{cccc} B_{11} & B_{12} & \cdots & B_{1r}\\ B_{21} & B_{22} & \cdots & B_{2r}\\ \vdots & \vdots & \ddots & \vdots \\ B_{r1} & B_{r2} & \cdots & B_{rr} \end{array}\right).$$

Similarly, the second term in Eq. 5 can be reformulated as

(9) $$\sum_{r,j=1}^{V}∥\psi_{v}-\psi_{k}{∥}_{2}^{2}=\sum_{r,j=1}^{V}{\left(P_{r}w_{r}-P_{j}w_{j}\right)}^{T}\left(P_{r}w_{r}-P_{j}w_{j}\right)\;=\sum_{r,j=1}^{V}\left(w_{r}^{T}P_{r}^{T}P_{r}w_{r}-w_{r}^{T}P_{r}^{T}P_{j}w_{j}-w_{j}^{T}P_{j}^{T}P_{r}w_{r}+w_{j}^{T}P_{j}^{T}P_{j}w_{j}\right)\;=\sum_{r=1}^{V}\sum_{j=1}^{V}w_{r}^{T}\left(P_{r}^{T}P_{r}+P_{j}^{T}P_{j}-2P_{r}^{T}P_{j}\right)w_{j}\;=\sum_{r=1}^{V}\sum_{j=1}^{V}w_{r}^{T}M_{rj}w_{j}\;=W^{T}MW,$$

where $P_{r}={\left(X_{r}^{T}X_{r}\right)}^{-1}X_{r}^{T}$, and

(10) $$M_{rj}=\left\{\begin{array}{c} 2(V-1)P_{r}^{T}P_{r}\;r=j\\ -2P_{r}^{T}P_{j}\;r\neq j \end{array}\right..$$

The multi-omics correspondence matrix M is defined by

(11) $$M=\left(\begin{array}{cccc} M_{11} & M_{12} & \cdots & M_{1r}\\ M_{21} & M_{22} & \cdots & M_{2r}\\ \vdots & \vdots & \ddots & \vdots \\ M_{r1} & M_{r2} & \cdots & M_{rr} \end{array}\right).$$

Substituting Eq. 6 and Eq. 9 into Eq. 5, the objective function of PANDA can be rewritten into the following equivalent form:

(12) $$\underset{W}{\text{argma}x\;}W^{T}BW-\eta W^{T}MW.$$

***Proposition 1:*** The objective function of PANDA as defined in Eq. 12 has a trivial solution of all zeros.

***Proof:*** If $\overset{‾}{W}$ is a solution to Eq. 12, then a nonzero constant k exists with $k^{2}<1$ such that $k\overset{‾}{W}$ is a better solution to Eq. 12. Now let $\delta (W)=W^{T}BW-\eta W^{T}MW$, we have

$$\delta (k\overset{‾}{W})=k^{2}{\overset{‾}{W}}^{T}B\overset{‾}{W}-\eta k^{2}{\overset{‾}{W}}^{T}M\overset{‾}{W}\;=k^{2}\left({\overset{‾}{W}}^{T}B\overset{‾}{W}-\eta {\overset{‾}{W}}^{T}M\overset{‾}{W}\right)\;\leq {\overset{‾}{W}}^{T}B\overset{‾}{W}-\eta {\overset{‾}{W}}^{T}M\overset{‾}{W}\;=\delta (\overset{‾}{W})$$

Therefore, when k→0, which implies $k\overset{‾}{W}\to 0$, we have $\delta (k\overset{‾}{W})\to {\overset{‾}{W}}^{T}B\overset{‾}{W}-\eta {\overset{‾}{W}}^{T}M\overset{‾}{W}$, and thus 0 is a trivial solution to the objective function in Eq. 12. ■

To avoid arbitrary scaling and the trivial solutions of all zeros, the transformation vectors of PANDA are constrained to be uncorrelated with respect to the cross-covariance matrices (i.e., $w_{r}^{T}X_{r}^{T}X_{v}w_{v}=1$ for $\left.r,v=1,\ldots ,V\right)$. As in Eq. 6 and Eq. 9, the uncorrelated constraint can be reformulated into the following compact form:

(13) $$\sum_{r=1}^{V}\sum_{j=1}^{V}w_{r}^{T}X_{r}^{T}X_{j}w_{j}=\sum_{r=1}^{V}\sum_{j=1}^{V}w_{r}^{T}X_{r}^{T}X_{j}w_{j}\;=\sum_{r=1}^{V}\sum_{j=1}^{V}w_{r}^{T}C_{rj}w_{j}\;=W^{T}CW,$$

where $C_{rj}=X_{r}^{T}X_{j}$ is the two-omics cross-covariance matrix. The multi-omics variance covariance matrix C is defined by

(14) $$C=\left(\begin{array}{cccc} C_{11} & C_{12} & \cdots & C_{1r}\\ C_{21} & C_{22} & \cdots & C_{2r}\\ \vdots & \vdots & \ddots & \vdots \\ C_{r1} & C_{r2} & \cdots & C_{rr} \end{array}\right).$$

Thus, the formulation of PANDA together with the uncorrelated constraint becomes

(15) $$\underset{W}{\text{argma}x\;}W^{T}BW-\eta W^{T}MW.$$

$$\text{s.t}\;W^{T}CW=\text{I}$$

The constraint in the objective function in Eq. 15 not only helps avoid the trivial solution and extract uncorrelated components but also helps PANDA gain more discriminative power, as the main diagonal elements of the matrix C are the total scatter matrices (i.e., the sums of the between- and within-class variations of the different omics). Also, the nondiagonal elements of matrix C help capture the interactions among the different omics. Thus, maximizing the objective function in Eq. 15 together with the constraint ensures that PANDA extracts discriminant, uncorrelated latent components that capture the cross-omics interactions (or correlations). The objective function in Eq. 15 can be further reduced to

(16) $$\underset{W}{\text{argma}x\;}W^{T}DW,$$

$$\text{s.t}\;W^{T}CW=\text{I}$$

where D=B-ηM. The Lagrangian associated with Eq. 16 is

(17) $$L\left(W,\Omega \right)=W^{T}DW-\Omega \left(W^{T}CW-I\right),$$

where Ω is the Lagrange multiplier matrix. Taking the derivative of the Lagrangian (Eq. 17) with respect to W and setting it to zero, we have

$$\frac{\partial L(W,\Omega )}{\partial W}=DW-\Omega CW=0.$$

Thus, we have

(18) DW=ΩCW.

The optimal transformation matrix $W^{*}$ can then be extracted via a generalized eigenvalue decomposition (Eq. 18).

The matrix C in the generalized eigenproblem (Eq. 18) is required to be nonsingular to obtain a stable solution of the generalized eigenvalue problem. To ensure that matrix C is nonsingular, we adopt the idea of regularization by adding a small constant value to the diagonal elements of C as C+αI for some α>0. It is easy to see that C+αI is nonsingular and that the transformation vectors that form the transformation matrix are extracted as the eigenvectors of the generalized eigenproblem

(19) $$DW=\Omega \left(C+\alpha I\right)W.$$

Eigenspaces are well known to be invariant under a diagonal shift. Thus, the generalized eigenvalue problems in Eq. 18 and Eq. 19 have the same solution.

After obtaining the transformation matrix $W=\left[W_{1},\ldots ,W_{v}\right]$, the uncorrelated discriminant latent components for each omics can be extracted as

(20) $$\begin{array}{c} Z_{1}=X_{1}W_{1}\\ \vdots \\ Z_{v}=X_{v}W_{v} \end{array}.$$

Owing to the discriminant and cross-omics correlation terms in the PANDA objective function, the extracted components of each omics datum (e.g., $Z_{1}$) captures as much cross-omics correlation and correspondence information from the different omics datasets as possible. The extracted PANDA components capture the discriminant structure in the original data, allowing for class separation in the low-dimensional inferred latent spaces. The entries of the loading matrices $W_{v}$ are weights/scores indicating the association of the original features (e.g., genes) with the extracted PANDA components, allowing for biological interpretation of the PANDA components. Features with strong associations with PANDA components will have larger absolute weights in the corresponding entries of $W_{v}$, whereas features with little to no association with the PANDA components will have values close to zero. The sign of the weights indicates the direction of the effects. The complete algorithm for PANDA is given in Algorithm 1.

**Algorithm 1:** PANDA

**Input:** The ν omics data matrices $X_{\nu }$ with labels $L=\left\{l_{1},\ldots ,l_{n}\right\}$ and regularization parameter η.
**Output:** Discriminative latent components $Z_{1},\ldots ,Z_{\nu }$, and optimal transformation matrices $W_{1},\ldots ,W_{\nu }$.
1. Construct the multi-omics between-class scatter matrix B as in Eq. 8.
2. Construct the multi-omics representation correspondence matrix M as in Eq. 11.
3. Construct the multi-omics total scatter matrix C as in Eq. 14.
4. Obtain the optimal transformation matrices $W_{1},\ldots ,W_{\nu }$ by solving the objective function in Eq. 16.
5. Extract the discriminative latent components $Z_{1},\ldots ,Z_{\nu }$ according to Eq. 20.

### Downstream analysis

The learned transformation (loading) matrices from the PANDA model are used to determinethe latent components for both the training and unseen samples as well as to provide insight into the biological meaning of the latent components. Based on the extracted latent components, four popular downstream analyses were performed: ordination of samples in latent spaces, gene set enrichment analysis, classification, and clinical outcome prediction. Low-dimensional representations of the data were visualized to examine the discriminant power of the latent components as well as the driving force for sample heterogeneity. The loading matrices provided weights used to identify the most important features and biological processes driving the heterogeneity captured by each latent component. Using the top gene sets, gene set enrichment analysis was performed using g:Profiler to ascertain biological meanings to the latent components. Using the breast cancer dataset from the mixOmics package, use of PANDA latent components to improve classification tasks was demonstrated. The latent components from the training data were used to train a classification model, and the predicted latent components forthe test samples were used to evaluate the performance of the classification model. Furthermore, the associations of the latent components with patients’ clinical outcomes were assessed using the Cox proportional hazards model.

### Benchmarking methods

For the simulated and real-world single-cell multi-omics data, the performance of PANDA was benchmarked against nine other multi-omics approaches for integrating multi-omics data and against Seurat, which is designed specifically for single-cell analysis. Specifically, the discriminant capability and 2D visualizations of the different methods were compared. For the TCGA datasets, the nine multi-omics integration approaches were further compared with PANDA in terms of discriminant feature extraction and clinical outcome prediction. For the single-cell data analysis, the preprocessed scRNA-seq and scATAC-seq data obtained after applying the standard Seurat preprocessing pipeline (i.e., normalization and differential variable feature selection) were used as inputs to the different multi-omics integration approaches. Detailed settings used in the benchmarked approaches are described below.

#### Seurat

Seurat^76^ is one of the most widely used single-cell multi-omics data integration tools. For our single-cell analysis using Seurat, the raw gene expression and count matrix of scRNA-seq data and unnormalized gene activity score matrix of scATAC-seq data were fed into Seurat, which was followed by data normalization using the *NormalizeData* function. The most highly variable genes were then selected using the *FindVariableFeatures* function, and the resulting variables were scaled and centered using the *ScaleData* function. The scATAC-seq data were processed by performing TF-IDF normalization on the count matrix using the *RunTFIDF* function. The top features in the scATAC-seq data were then identified using the *FindTopFeatures* function, and the retained variables were centered and scaled using the *ScaleData* function. To integrate the scRNA-seq data with the scATAC-seq, the anchors between these two datasets were identified using the *FindTransferAnchors* function with “CCA” as the dimension reduction method, and the *TransferData* function was used to transfer data from scRNA-seq to scATAC-seq.

#### iCluster

iCluster^15^ is another well-known multi-omics integrative model that determines a joint latent component Z that connects the V different omics:

(21) $$\begin{array}{c} X_{1}=W_{1}Z+\varepsilon_{1}\\ X_{2}=W_{2}Z+\varepsilon_{2}\\ \vdots \\ X_{V}=W_{V}Z+\varepsilon_{V} \end{array}.$$

A likelihood-based solution to the above formulation (Eq. 21) is obtained based on the assumptions that $Z^{*}\sim N(0,I)$ and ε~N(0,Ψ), where $\Psi =\text{diag}\left(\psi_{1},\ldots ,\psi_{\sum_{v}n_{v}}\right)$ is a diagonal covariance matrix and $Z^{*}$ is a continuous parameterization of Z. The expectation-maximization algorithm is used to obtain the maximum likelihood estimates of the matrices $W={\left[W_{1},\ldots ,W_{V}\right]}^{T}$ and Ψ. The log-likelihood function of the data is given by

(22) $$l\left(W,\Psi \right)=-\frac{m}{2}\left(\sum_{v=1}^{V}n_{v}In\left(2\pi \right)+In\text{det}\left(\Psi \right)\right)-\frac{1}{2}\left(tr\left({\left(X-WZ^{*}\right)}^{T}\Psi^{-1}\left(X-WZ^{*}\right)\right)+tr\left(Z^{*T}Z^{*}\right)\right).$$

To obtain a sparse coefficient matrix W, which is very important for variable selection, a penalty term with a nonnegative constraint is added to the log-likelihood formulation (Eq. 22) as

(23) $$l\left(W,\Psi \right)=-\frac{m}{2}\left(\sum_{v=1}^{V}n_{v}In\left(2\pi \right)+\text{In}\;\text{det}\left(\Psi \right)\right)-\frac{1}{2}(\text{tr}\left({\left(X-WZ^{\text{*}}\right)}^{T}\Psi^{-1}\left(X-WZ^{\text{*}}\right)\right)+\text{tr}\left(Z^{\text{*}T}Z^{\text{*}}\right))-\gamma \sum_{v=1}^{V}\sum_{k=1}^{K-1}\sum_{j=1}^{n_{v}}\left|w_{vkj}\right|.$$

After obtaining the component matrix Z, the K-means algorithm is applied to the component matrix to recover the data subtypes. Owing to the sparse solutions of W, the coefficients of some of the variables are shrunk to zero, which helps highlight which variables are important in identifying the different data subtypes. iCluster was implemented using the iCluster package available at https://rdrr.io/bioc/iClusterPlus/man/iCluster.html.

#### tICA

Our PANDA method was also benchmarked with tensor-based multi-omics data integration approach tICA^14^, which incorporates tensor and independent component analysis^77^ to integrate multi-omics datasets with the aim of identifying sources of variation in the data. Because this approach requires the different data omics to have the same samples and features, an approach similar to that described by Cantini et al.^6^ was used by computing correlation matrices common to the different omics data. For tICA, the different omics data matrices $X_{v},v=1,2,\ldots ,V$ are structured into an order-3 data tensor $\underline{X}$ of dimension $d_{1}\times d_{2}\times d_{3}$. tICA then determines a decomposition of the form

(24) $$\underline{X}=J\;{☉}_{v=1}^{V}\Omega_{v},$$

where J is an order-3 tensor of dimension $d_{1}\times d_{2}\times d_{3}$ composed of mutually statistically independent random variables $j_{1},j_{2},\ldots ,j_{V}$ satisfying $E\left(j_{1},j_{2},\ldots ,j_{V}\right)=0$ and $Var\left(j_{1},j_{2},\ldots ,j_{V}\right)=I,\Omega_{v}$ is a $d_{m}\times d_{m}$ matrix, and ⊙ denotes the tensor contraction operator. Eq. 24 can be solved using several approaches. In our analysis, the fourth-order blind identification^78^ approach was used, which maximizes the fourth-order moments (kurtosis) of the independent components $j_{1},j_{2},\ldots ,j_{V}$. More details about the fourth-order blind identification approach were reported by Virta et al.^79^. tICA was implemented in our study using the tensorICA package, which available at https://rdrr.io/github/jinghan1018/tensorICA/man/DoTICA.html.

#### MCIA

MCIA^10–12^ is another integrative analysis algorithm aimed at capturing the common relationships as well as assessing the concordance among multi-omics datasets. MCIA finds a set of loading vectors $w_{v}\in R^{n_{v}}$ and a vector u that maximizes the objective function

(25) $$\underset{w_{1},\ldots ,w_{V}}{\text{ma}x}\sum_{v=1}^{V}s_{v}{\left(u^{T}DX_{v}Q_{v}w_{v}\right)}^{2},$$

$$\text{s.t}\;w_{v}^{T}Q_{v}w_{v}=1,v=1,\ldots ,V,u^{T}Du=1,$$

where $s_{v}$ is a positive weight for the vth omics data such that $\sum s_{v}=1,D\in R^{m\times m}$ is a diagonal matrix designed to put strong emphasis on certain samples by assigning larger weights to the corresponding diagonal entries of D and $Q_{v}\in R^{n_{v}\times n_{v}}$ is also a diagonal matrix that can be designed in such a way that highly variable features have larger weights in the corresponding entries of $Q_{v}$. The vector u in the objective function (Eq. 25) can be extracted as the eigenvector solution to the following eigenvalue problem:

(26) $$\hat{X}\hat{Q}{\hat{X}}^{T}Du=\lambda u,$$

where $\overset{ˆ}{X}=\left[s_{1}^{1/2}X_{1},s_{2}^{1/2}X_{2},\ldots ,s_{v}^{1/2}X_{v}\right]\in R^{m\times \sum n_{v}}$ is the concatenated data matrix of the V weighted omics datasets and $\overset{ˆ}{Q}$ is a block diagonal matrix whose diagonal blocks are $Q_{1},\ldots ,Q_{V}$. After extracting the vector u from Eq. 26, the loading vectors $w_{k}$ are obtained by

(27) $$w_{k}=\frac{X_{v}^{T}Du}{{∥X_{v}^{T}Du∥}_{Q_{v}}},$$

where $∥\cdot {∥}_{Q_{v}}$ denotes the $Q_{v}$-normed operator. The subsequent sets of loadings can be extracted by repeating the procedure described above with the residual matrices computed by deflating^80^ the omics datasets. The R implementation of MCIA from the omicade4 package was used in our benchmarking analysis.

#### RGCCA

RGCCA^2^ is an extension of the famous two-view canonical correlation analysis (CCA)^81^ to handle multiple-view (>2) datasets. RGCCA extracts information shared by the V omics datasets considering an a priori graph connecting the different omics datasets. Given a design matrix $G=\left\{g_{vk}\right\}$, where $g_{vk}=1$ if the two omics $X_{v}$ and $X_{k}$ are connected and $g_{vk}=0$, otherwise, the objective function of RGCCA can be formulated as

(28) $$\underset{w_{1},\ldots ,w_{V}}{\text{ma}x}\sum_{v,k=1;v\neq k}^{V}g_{vk}f\left(cov\left(X_{v}w_{v},X_{k}w_{k}\right)\right),$$

$$\text{s.t}\;\left(1-\tau_{v}\right)var\left(X_{v}w_{v}\right)+\tau_{v}{∥w_{v}∥}_{2}^{2}=1,v=1,\ldots ,V,$$

where $\tau_{1},\ldots ,\tau_{v}$ are shrinkage constants in [0,1] and f may be defined as f(x)=x, or $f(x)=x^{2}$. The objective function in Eq. 28 does not have a known analytical solution; thus, the partial least squares (PLS)^82^ algorithm is usually employed to optimize the objective function in Eq. 28. PLS starts with an arbitrary choice of normalized weight vector $w_{1}^{0},w_{2}^{0},\ldots ,w_{V}^{0}$. Assuming the weight vectors $w_{1}^{s+1},w_{2}^{s+1},\ldots ,w_{V}^{s+1}$, and s=0,1,… have been computed, to compute the weight vector $w_{v}^{s+1}$, an inner component $r_{v}^{s}$ is first computed by

(29) $$r_{v}^{s}=\sum_{k<v}g_{vk}f(cov\left(X_{v}w_{v}^{s},X_{k}w_{k}^{s+1}\right)X_{k}w_{k}^{s+1}+\sum_{k>v}g_{vk}f(\text{cov}\left(X_{v}w_{v}^{s},X_{k}w_{k}^{s}\right)X_{k}w_{k}^{s}.$$

The weight vector $w_{v}^{s+1}$ is then computed by

(30) $$w_{v}^{s+1}={\left[{\left(r_{v}^{s}\right)}^{T}X_{v}{\left[\tau_{v}I+\left(1-\tau_{v}\right)\frac{1}{n}X_{v}^{T}X_{v}\right]}^{-1}X_{v}^{T}r_{v}^{s}\right]}^{1/2}{\left[\tau_{v}I+\left(1-\tau_{v}\right)\frac{1}{n}X_{v}^{T}X_{v}\right]}^{-1}X_{V}^{T}r_{v}^{s}.$$

This procedure is repeated iteratively until convergence. After computing the weight vectors $w_{v}$, the RGCCA outer components for the vth omics data are then computed as

$$z_{v}=X_{v}w_{v}.$$

The R implementation of the RGCCA algorithm available at https://rdrr.io/cran/RGCCA/man/rgcca.html was used in our benchmarking analysis.

#### intNMF

intNMF^1^ is an integrative algorithm based on classical nonnegative matrix factorization (NMF)^83,84^ that determines a factorization of a matrix $X\in R^{m\times n}$ having all nonnegative entries into two nonnegative matrices such that

(31) X≈WH,

where $W\in R^{m\times d}$ and $H\in R^{d\times n}$ are the basis and coefficient matrices, respectively. intNMF is an extension of NMF to handle multi-omics datasets. This method operates on the V omics datasets to determine a common basis matrix W and omics-specific coefficient matrices $H_{v}$ such that

(32) $$\begin{array}{c} X_{1}\approx WH_{1}\\ X_{2}\approx WH_{2}\\ \vdots \\ X_{v}\approx WH_{v} \end{array},$$

where the matrices W and $H_{v}$ are nonnegative. The objective function of the intNMF algorithm is given by

(33) $$\underset{W,H}{\text{min}}\sum_{v=1}^{V}\delta^{v}{\left\|X_{v}-WH_{v}\right\|}_{2},$$

where $\delta^{v}>0$ is a user-specified weight for the vth omics data. The objective function (Eq. 33) is nonconvex with respect to W and $H_{v}$ together. To overcome this issue, a nonnegativity constrained least squares^85^ algorithm is usually used to solve for W and $H_{v}v=1,2,\ldots ,V$ in the intNMF objective function (Eq. 33). The idea here is that the nonconvex objective function can be converted into a convex one in W given $H_{v}$ to solve for W and vice versa. This entire approach can be summarized into three steps. First, the matrix W is randomly initialized, usually from a uniform distribution U~[0,1]. Second, utilizing the obtained W, the individual matrices $H_{v}v=1,2,\ldots ,V$ are obtained by solving the following objective function using the nonnegativity constrained least squares algorithm:

(34) $$\underset{H_{v}}{\text{min}}{\left\|X_{v}-WH_{v}\right\|}_{2}.$$

$$\text{s.t}\;H_{v}\geq 0$$

Third, using the solutions for $H_{v}$ in Eq. 34, the nonnegativity constrained least squares algorithm is used again to solve for W by optimizing the following objective function:

(35) $$\underset{W}{\text{min}}\sum_{v=1}^{V}\delta^{v}{\left\|X_{v}-WH_{v}\right\|}_{2}.$$

s.tW≥0

This procedure is repeated until convergence. The obtained matrix W is common across the different V omics datasets and contains cluster structures from all of the datasets. After computing the matrices W and $H_{v}$, samples in the datasets are assigned to clusters based on the entries of W, where larger weights in the columns of W indicate the cluster to which samples are associated with. The R implementation of the intNMF algorithm from the intNMF R package was used for our benchmarking analysis.

#### MOFA

MOFA^8,9^ is a generalization of the traditional factor analysis^86^ model aimed at reducing the dimensionality of data while capturing the correlation among the data variables as well as the variance of the variables. Unlike factor analysis that works with a single omics dataset, MOFA is designed to handle V(V>1) omics datasets and determines a factorization

(36) $$X_{gv}=W_{v}Z_{g}+\varepsilon_{gv},\;v=1,\ldots ,V,g=1,\ldots ,G,$$

where $X_{gv}$ denotes the data matrix for the vth omics data and the gth group, $W_{v}$ denotes the loadingmatrix for the vth omics data, $Z_{g}$ denotes the shared factor matrix containing the latent vectors for the gth group, and $\varepsilon_{gv}$ denotes the residual noise for the vth omics data and the gth group. MOFA uses a probabilistic Bayesian approach to assigning prior distributions on the factors $Z_{g}$, on the loadings $W_{v}$, and on the residual noise $\varepsilon_{gv}$. The spike-and-slab prior^87^ with a reparameterization of the weights is used to encode sparsity on the loadings by forcing some of the weights and factors to zero:

(37) $$p\left({\hat{w}}_{v}^{kd},s_{v}^{kd}\right)=𝒩\left({\hat{w}}_{v}^{kd}|0,\frac{1}{\alpha_{v}^{k}}\right)Ber\left(s_{v}^{kd}|\theta_{v}^{k}\right)\;p\left(\theta_{v}^{k}\right)=ℬ\left(\theta_{v}^{k}|a_{0}^{\theta },b_{0}^{\theta }\right)\;p\left(\alpha_{v}^{k}\right)=𝒢\left(\alpha_{v}^{k}|a_{0}^{\alpha },b_{0}^{\alpha }\right),$$

where $s_{v}^{kd}$ denotes a Bernoulli random variable for omics data v, feature d, and factor $k;\alpha_{v}^{k}$ controls the strength of factor k in omics data v; $\theta_{v}^{k}$ controls the sparsity levels of factor k in omics data v;𝒩(x∣μ,σ) implies x follows a normal distribution with mean μ and SD σ;Ber(x,θ) implies x follows a Bernoulli distribution with parameter θ;ℬ(x∣a,b) implies x follows a beta distribution with shape and rate parameters a and b; and 𝒢(x∣a,b) implies x follows a gamma distribution with shape and rate parameters a and b. MOFA also uses a prior that captures structural sparsity where some factors are allowed to be active and/or captures variability in different subsets of groups:

(38) $$p\left({\hat{z}}_{g}^{nk},s_{g}^{nk}\right)=𝒩\left({\hat{z}}_{g}^{nk}|0,\frac{1}{\alpha_{g}^{k}}\right)Ber\left(s_{g}^{nk}|\theta_{g}^{k}\right)\;p\left(\theta_{g}^{k}\right)=ℬ\left(\theta_{g}^{k}|a_{0}^{\theta },b_{0}^{\theta }\right)\;p\left(\alpha_{g}^{k}\right)=𝒢\left(\alpha_{g}^{k}|a_{0}^{\alpha },b_{0}^{\alpha }\right),$$

where $s_{g}^{nk}$ denotes a Bernoulli random variable for group g, sample n, and factor k. Because different sample groups may present different noise levels, MOFA assigns the following prior to the noise residuals:

$$p\left(\varepsilon_{gv}^{r}\right)=𝒩\left(\varepsilon_{gv}^{r}|0,\frac{1}{\tau_{gv}^{r}}\right)\;p\left(\tau_{gv}^{r}\right)=𝒢\left(\tau_{gv}^{r}|a_{0}^{\tau },b_{0}^{\tau }\right),$$

where $\varepsilon_{gv}^{r}$ denotes the noise residual for feature r in omics data v and group g. Benefitting from the use of these priors, MOFA can integrate multi-omics datasets at both the feature and sample level by extracting factors associated with few active and/or important features.

#### MEFISTO

MEFISTO^3^ is an extension of the MOFA model to handle multimodal data with continuous structures (e.g., temporal or spatial relationships). MEFISTO uses the continuous sample relationship to guide the integration of multimodal data while capturing changes in time and/or space variation from other sources of variation that are independent of time and/or space. Specifically, each sample n in group g is characterized by a continuous covariate $c_{g}^{n}\in R^{q}$, which could be a time point (1D vector) or sample position in space (2D vector). Similar to MOFA, MEFISTO determines a decomposition of the form

(39) $$X_{gv}=W_{v}Z_{g}+\varepsilon_{gv},\;v=1,\ldots ,V,g=1,\ldots ,G,$$

where $X_{gv}$ denotes the data matrix for the vth omics data and the gth group, $W_{v}$ denotes the loading matrix for the vth omics data, $Z_{g}$ denotes the shared factor matrix containing the latent vectors for the gth group, and $\varepsilon_{gv}$ denotes the residual noise for the vth omics data and the gth group. The factor values $z_{g}^{n}$ are modeled via a latent process as

(40) $$p\left(z_{g}^{nk}|f_{k}\right)=f_{k}\left(c_{g}^{n}\right)+\eta_{g}^{nk}$$

with

$$p\left(f_{k}\right)\sim GP\left(f_{k}|0,\kappa^{k}\right)\;p\left(\eta_{g}^{nk}\right)\sim 𝒩\left(0,\zeta^{k}\right),$$

where GP(x∣μ,κ) implies that x follows a Gaussian process distribution with mean function μ and covariance function κ. The covariance function κ is defined using the squared exponential kernel

(41) $$\kappa^{k}\left(c_{g}^{n},c_{g^{\prime }}^{n^{\prime }}\right)=s^{k}\;\text{exp}\left(-\frac{{\left\|c_{g}^{n}-c_{g^{\prime }}^{n^{\prime }}\right\|}_{2}^{2}}{2{\left(\ell^{k}\right)}^{2}}\right)\kappa_{k}^{G}\left(g,g^{\prime }\right)$$

with

$$\kappa_{k}^{G}\left(g,g^{\prime }\right)=b^{k}{\left(b^{k}\right)}^{T}+\sigma^{k}I,b^{k}\in R^{G\times Q}\;\text{and}\;s^{k}=1-\zeta^{k},$$

where parameter $\ell^{k}$ controls the rate at which the sample correlation decays along the covariate, and parameter $s^{k}=1-\zeta^{k}$ controls the proportion of smooth variation captured by the factor k, and the hyperparameters $b^{k}$. As in the original MOFA model, the spike-and-slab prior is utilized used to encode sparsity on the loadings matrix $W_{v}$, and the following prior distribution is used for the factors k:

(42) $$p\left(z_{:}^{k}\right)=𝒩\left(z_{:}^{k}|0,\Sigma^{k}\right)$$

with

$${\left(\Sigma^{\text{k}}\right)}_{nn^{\prime }}=\left(1-\zeta^{k}\right)\text{exp}\left(-\frac{∥c_{g}^{n}-c_{g^{\prime }}^{n^{\prime }}{∥}_{2}^{2}}{2{\left(\ell^{k}\right)}^{2}}\right)\kappa_{k}^{G}\left(g,g^{\prime }\right)+\zeta^{k}\delta^{nn^{\prime }}.$$

#### DIABLO

DIABLO^4^ extends the unsupervised classical regularized and sparse generalized canonical correlation analyses^2,88^ to a supervised framework where multi-omics data are integrated by finding lower dimensional subspaces that discriminate the different phenotypic groups in the data. The classical sparse generalized canonical correlation analysis method determines projections that maximize the correlation among the extracted components by solving the following objective function:

(43) $$\underset{w_{1},\ldots ,w_{V}}{\text{ma}x}\sum_{v,k=1;v\neq k}^{V}g_{vk}cov\left(X_{v}w_{v},X_{k}w_{k}\right),$$

$$\text{s.t}\;∥w_{v}{∥}_{2}=1,\;\text{and}\;∥w_{v}{∥}_{1}\leq \lambda^{v}\;\text{for}\;v=1,\ldots ,V$$

where $G=\left\{g_{vk}\right\}$ is a V×V design matrix similar to the one in Eq. 28 and $\lambda^{v}$ is a nonnegative parameter that controls the sparsity in the loading vector $w_{v}$. DIABLO extends sparse generalized canonical correlation analysis to a supervised framework by substituting one of the omics data matrices in Eq. 43 with a class membership matrix Y similar to the one defined in Eq. 2. The design matrix G is determined using either prior biological knowledge or a data-driven approach. Two possible ways of constructing the design matrix G are the null design

(44) $$G_{\text{null}}=\left(\begin{array}{lll} 0 & 0 & 0\\ 0 & 0 & 0\\ 0 & 0 & 0 \end{array}\right),$$

where all entries of the design matrix are zeros, indicating none of the omics datasets are connected; and the full design

(45) $$G_{\text{full}\;}=\left(\begin{array}{lll} 0 & 1 & 1\\ 1 & 0 & 1\\ 1 & 1 & 0 \end{array}\right),$$

where the off-diagonal entries are all equal to one, indicating that all omics datasets are connected. The entries of the design matrix determine a trade-off between correlation and discrimination. With the design matrix defined as in Eq. 44, DIABLO focuses on extracting discriminant components and disregards the correlation among the different omics datasets, whereas with G defined as in Eq. 45, DIABLO focuses on extracting components that capture the correlation among the different omics datasets. In general, DIABLO prioritizes the correlation among the different omics for values from 0.5 to 1 in the design matrix, whereas for values below this range, DIABLO prioritizes capturing the discriminant information in the data. The DIABLO latent components are extracted using the partial least squares approach similar to the one described above in the RGCCA section.

#### JIVE

JIVE^13^ is another multi-omics integrative algorithm aimed at separating the joint and individual structures and/or effects in multi-omics datasets. JIVE determines decomposition of variations in multi-omics datasets into low-rank approximations capturing joint variations across omics, low-rank approximations capturing individual structured variations specific to each omics dataset and residual noise. Mathematically, JIVE determines a decomposition of the form

(46) $$\begin{array}{c} X_{1}=J_{1}+A_{1}+\varepsilon_{1}\\ X_{2}=J_{2}+A_{2}+\varepsilon_{2}\\ \vdots \\ X_{V}=J_{V}+A_{V}+\varepsilon_{V} \end{array},$$

where $\varepsilon_{v}$ represents the residual matrix associated with $X_{v}$ and the matrices $A_{v}$ and $J_{v}$ represent the individual and joint structures associated with $X_{v}$, respectively. JIVE enforces rank and orthogonality constraints to uniquely extract the individual and joint components. Let

(47) $$R=\left[\begin{array}{c} R_{1}\\ R_{2}\\ \vdots \\ R_{V} \end{array}\right]=\left[\begin{array}{c} X_{1}-J_{1}-A_{1}\\ X_{2}-J_{2}-A_{2}\\ \vdots \\ X_{V}-J_{V}-A_{V} \end{array}\right]$$

represent the matrix of residuals after accounting for the individual and joint structures. The matrices J and $A_{v}$ are computed through minimization of $∥R{∥}^{2}$ using rank constraints rank(J)=r and $\text{rank}\left(A_{v}\right)=r_{v}$, where

(48) $$J=\left[\begin{array}{c} J_{1}\\ J_{2}\\ \vdots \\ J_{V} \end{array}\right].$$

The JIVE algorithm starts by initializing J and $A_{v}$ using the initial ranks for the joint and individual structures and proceed to computes a new estimate for the rank of the joint structure after deflating the combined data matrix, X-A, where

(49) $$X=\left[\begin{array}{c} X_{1}\\ X_{2}\\ \vdots \\ X_{V} \end{array}\right]\;\text{and}\;A=\left[\begin{array}{c} A_{1}\\ A_{2}\\ \vdots \\ A_{V} \end{array}\right].$$

Similarly, the ranks of the individual structures are re-estimated after deflating the combined matrix by removing the already extracted joint structure X-J. This procedure is repeated until convergence.

### Data sets and preprocessing

#### Simulated data

Single-cell datasets consisting of bulk RNA-seq and DNase sequencing profiles from the same samples were simulated using the MOSim R package available at https://rdrr.io/bioc/MOSim/man/mosim.html. Specifically, two data matrices were constructed as

$$X_{1}=W^{\left(1\right)}H$$

$$X_{2}=W^{\left(2\right)}H,$$

where

$$W_{ij}^{\left(1\right)}=\left\{\begin{array}{c} 1,1+x_{j}(200)\leq i\leq 200+n_{j}(200)\\ 0,\;\text{otherwise} \end{array}\right.$$

$$W_{ij}^{\left(2\right)}=\left\{\begin{array}{c} 1,1+x_{j}(500)\leq i\leq 500+n_{j}(500)\\ 0,\;\text{otherwise} \end{array}\right.$$

$$H_{kj}=\left\{\begin{array}{c} 1,1+x_{k}(c)\leq j\leq c+x_{k}(c),k\leq K-1\;\text{or}\;1+x_{k}(c)\leq i\leq n,k=K\\ 0,\;\text{otherwise} \end{array}\right.$$

with $x_{j}(n)=(j-1)(n-\text{coph}),\;\text{coph}\;=0$, and K denotes the maximum between $K_{1}$ (rank of $\left.W^{\left(1\right)}\right)$ and $K_{2}$ (rank of $W^{\left(2\right)}$). The parameter $K_{1}$ is varied from 3 to 7, and $K_{2}$ is fixed to 3, resulting in clusters that were defined from gene expression and not reflecting epigenetic distinctions. Also, Gaussian noises were added to $W^{\left(1\right)}$ and $W^{\left(2\right)}$, and negative entries of these matrices were set to zero. To evaluate the robustness of the different methods with very sparse and noisy datasets, as is the case with single-cell and most multi-omics datasets, dropouts and Gaussian noises were further added to both $X_{1}$ and $X_{2}$. PANDA was compared with the benchmarked integrative multi-omics approaches in terms of capturing the discriminant structures defined according to gene expression and was examined whether strong noise and large sparsity levels affect its performance. More details regarding the simulated data were described by Jin et al.^89^.

#### Single-cell cancer cell line data

The paired scRNA-seq and scATAC-seq datasets^16^ simultaneously measuring gene expression and chromatin accessibility on three cancer cell lines (HTC, HeLa, and K562) for a total of 206 cells were obtained from Cantini et al.^6^. The datasets were preprocessed by normalizing and scaling them. Next, variable gene and peak selection (top 1500) using a statistical test of the associations among the variables and cancer cell lines. Furthermore, the g:Convert function in the gprofiler2 package in R was used to convert Ensembl IDs to gene symbols.

#### Multiome PBMC data

Paired scRNA-seq and scATAC-seq datasets containing 11,909 cells, available as part of the SeuratData R package and originally hosted at the 10x Genomics website (https://support.10xgenomics.com/single-cell-multiome-atac-gex/datasets/1.0.0/pbmc_granulocyte_sorted_10k), were downloaded and used for our analysis. Quality control for the scRNA-seq data was conducted by filtering cells having more than 1000 and fewer than 25,000 unique molecular identifiers and with fewer than 20% mitochondrial transcripts. Similarly, the scATAC-seq data were filtered by retaining cells with more than 5000 and fewer than 70,000 total counts. After initial filtering, the features in both the scRNA-seq and scATAC-seq datasets were scaled and centered, and the 1500 most highly variable genes were selected via a statistical test of the associations among the features and cell types. The retained features were then used for integrative analysis.

#### Breast cancer data

TCGA breast cancer data were obtained using the mixOmics^50^ package. The data were already preprocessed and contained mRNA, miRNA, and protein expression levels measured for the same samples and classified into three subtypes: luminal, Her2 and basal. The training sets were used to train the different multi-omics integration models, and the test sets were used to evaluate the performance of the different models.

#### TCGA cancer multi-omics datasets

The five TCGA cancer datasets (GBM, breast, colon, kidney, and lung cancer) analyzed by Wang et al.^90^ were downloaded from http://compbio.cs.toronto.edu/SNF/SNF/Software.html. These datasets contain mRNA, miRNA, and methylation expression of samples from the same cohort. An approach similar to that described by Singh et al.^4^ was used to split the samples into two groups: high- and low-risk groups. Because these datasets potentially contain highly redundant features that may degrade the performance of the models, a chi-square test was used to select the top variable features associated with clinical outcomes.

### Evaluation metrics

To determine how well the class structures reflecting biological processes are captured in the low-dimensional latent spaces determined by the different integrative multi-omics methods we compared, consensus clustering was performed using a hierarchical clustering algorithm on the 2D latent space for each method. The ARI^91^ and NMI metrics^92,93^ were used to evaluate the similarity between the ground-truth clustering labels and the clustering labels obtained after performing clustering on the inferred 2D latent spaces. Greater ARI and NMI values indicated greater similarity between ground-truth labels and clustering labels inferred by the various methods, which in turn meant better accuracy. Let $C=\left\{C_{1},\ldots ,C_{l_{g}}\right\}$ and $C^{\prime }=\left\{C_{1}^{\prime },\ldots ,C_{l_{p}}^{\prime }\right\}$ denote the ground-truth and predicted cluster sets, respectively. The ARI can be computed by

ARI=n2(a+d)-[(a+b)(a+c)+(c+d)(b+d)]n22-[(a+b)(a+c)+(c+d)(b+d)],

where a denotes the number of pairs of objects in the same cluster in both C and $C^{\prime },b$ denotes the number of pairs of objects in the same cluster in C but in different clusters in $C^{\prime },c$ denotes the number of pairs of objects in different clusters in C but in the same cluster in $C^{\prime }$, and d denotes the number of pairs of objects in different clusters in both C and $C^{\prime }$.

The NMI is computed by

$$NMI=\frac{MI\left(C,C^{\prime }\right)}{\text{max}{\left(H(C),H\left(C^{\prime }\right)\right)}^{\prime }}$$

where $MI\left(C,C^{\prime }\right)$ denotes the mutual information estimate between C and $C^{\prime }$ and is defined by

$$MI\left(C,C^{\prime }\right)=\sum_{C_{i}\in C,C_{j}^{\prime }\in C^{\prime }}p\left(C_{i},C_{j}^{\prime }\right)\cdot {\text{log}}_{2}\frac{p\left(C_{i},C_{j}^{\prime }\right)}{p\left(C_{i}\right)\cdot p{\left(C_{j}^{\prime }\right)}^{\prime }}$$

where $p\left(C_{i}\right)$ and $p\left(C_{j}^{\prime }\right)$ denote the probabilities that a sample randomly selected from the data belongs to clusters $C_{i}$ and $C_{j}^{\prime }$, respectively, and $p\left(C_{i},C_{j}^{\prime }\right)$ denotes the joint probability that the randomly selected sample belongs to clusters $C_{i}$ and $C_{j}^{\prime }$, simultaneously. H(C) and $H\left(C^{\prime }\right)$ are the entropies of C and $C^{\prime }$, respectively, with

$$H(C)=-\sum_{C_{i}\in C}p\left(C_{i}\right)\cdot {\text{log}}_{2}\frac{p\left(C_{i}\right)}{n}$$

$$H\left(C^{\prime }\right)-\sum_{C_{j}^{\prime }\in C^{\prime }}p\left(C_{j}^{\prime }\right)\cdot {\text{log}}_{2}\frac{p\left(C_{j}^{\prime }\right)}{n},$$

where n denotes the total number of data samples. The values for ARI and NMI ranged from 0 to 1, with a value of 1 indicating that two sets of clusters are identical and a value of 0 indicating that two sets are independent.

Also, cluster purity^94^ was used to quantitatively evaluate cluster separation and consistency representation as determined by PANDA. Well-separated clusters reveal little to no overlap of samples belonging to different groups, thus attaining high purity values close to 1. The cluster purity is computed by

$$\text{Purity}=\frac{1}{n}\sum_{q=1}^{l_{p}}\underset{1\leq j\leq l_{g}}{\text{max}}\left(n_{q}^{j}\right),$$

where n denotes the total number of data samples and $n_{q}^{j}$ denotes the number of data samples in cluster q that belong to the ground-truth cluster $j\left(1\leq j\leq l_{g}\right)$.

### Gene set enrichment analysis and enrichment map of biological processes

For biomarker identification analysis, the PANDA latent components were extracted, and the most important features with the largest weights were identified. Enriched terms were identified using g:Profiler based on the 30 genes with the largest weights along the latent components. To account for multiple testing, the resulting P-values were adjusted using the Benjamini-Hochberg procedure^95^, and significant enrichments were reported at a false discovery rate of 5%. Enrichment map visualizations (performed using Cytoscape^96^) were also used to organize enriched biological processes in a network to provide a more concise and meaningful overview of these processes as characterized by the enriched marker genes. A false discovery rate Q-value of up to 0.05 was used for node filtering, and an edge cutoff (similarity) of 0.7 was used to identify similarity between gene pathways. Gene set enrichment analysis helped highlight the biological meaning of the PANDA latent components. Utilizing the g:Profiler we can identify biological processes and functions such as GO Biological Process, KEGG, and REACTOME pathways that are significantly enriched along the latent components. The identified gene markers were very diverse in terms of enriched biological processes.

### Survival analysis

The association of the PANDA latent components (and/or clustering results based on the latent components) with clinical covariates in the TCGA datasets was assessed using a Cox proportional hazards model. For a univariate association test based on the clustering results for the different benchmarked methods, a univariate Cox proportional hazards model was built, and the Harrell’s concordance indices of the different methods were compared. Similarly, univariate Cox proportional hazards models were built for the individual latent components of the benchmarked methods, and their predictive power was compared. For a multivariate test, the first 10 latent components of the different benchmarked methods were included as covariates in multivariate Cox proportional hazards models. The association of the first latent component of each method with survival was further investigated using Kaplan-Meier plots, in which the maximally selected rank statistics was used to determine the optimal cut point for each latent component.

## Extended Data

## Figures and Tables

**Table 1 |:** Summary of datasets

Dataset	Modality	Number of classes
**Simulated data**	scRNA-seq and scATAC-seq	5
**Cancer cell lines**	scRNA-seq and scATAC-seq	3
**PBMC10K**	scRNA-seq and scATAC-seq	19
**TCGA** b**reast cancer (mixOmics)**	mRNA, miRNA, and protein	3
**TCGA GBM**	mRNA, miRNA, and methylation	2
**TCGA** b**reast** cancer	mRNA, miRNA, and methylation	2
**TCGA** c**olon** cancer	mRNA, miRNA, and methylation	2
**TCGA** k**idney** cancer	mRNA, miRNA, and methylation	2
**TCGA** l**ung** cancer	mRNA, miRNA, and methylation	2

**Table 2 |:** Characteristics of the different benchmarked multi-omics integration algorithms

Multi-omics integration method	Captures discriminant structures	Captures cross-omics correlation	Captures omics correspondence/consistency	Determines omics-specific latent spaces (co-visualization)	Fast and scalable	Does not require domain knowledge	Corrects for technical variations
Seurat	**~**	✓	**X**	✓	**~**	**~**	✓
iCluster	**X**	✓	**~**	**X**	**X**	✓	**X**
Jive	**X**	**~**	**X**	**X**	**X**	✓	**X**
tICA	**X**	✓	**X**	✓	**X**	✓	**X**
MCIA	**X**	✓	**~**	**X**	✓	✓	**X**
RGCCA	**~**	✓	**X**	✓	✓	✓	**X**
intNMF	**X**	**~**	**X**	**X**	✓	✓	**X**
MOFA	**X**	**~**	**X**	**X**	**X**	✓	**~**
MEFISTO	**X**	**~**	**X**	**X**	**X**	✓	**~**
DIABLO	✓	✓	**~**	✓	✓	**X**	**X**
PANDA	✓	✓	✓	✓	✓	✓	✓

✓ Fulfills the criterion.

X Does not fulfill the criterion.

~ Partially fulfills the criterion.

## Data Availability

All datasets used in this study are publicly available. 1) The simulated single-cell multi-omics dataset was downloaded from https://github.com/squin/scAl. The single-cell multi-omics cancer cell line dataset was downloaded from https://github.com/cantinilab/momix-notebook. The PBMC multi-omics dataset was obtained from the Seurat package. The breast cancer multi-omics data used for breast cancer biomarker identification was obtained from the mixOmics package accessible at http://mixomics.org/mixdiablo/diablo-tcga-case-study/. The colon, breast, kidney, lung and GBM cancer datasets used for clinical outcome prediction were downloaded from http://compbio.cs.toronto.edu/SNF/SNF/Software.html.
